# Retrospective Proteomic Screening of 100 Breast Cancer Tissues

**DOI:** 10.3390/proteomes5030015

**Published:** 2017-07-07

**Authors:** Ida Pucci-Minafra, Gianluca Di Cara, Rosa Musso, Patrizia Cancemi, Nadia Ninfa Albanese, Elena Roz, Salvatore Minafra

**Affiliations:** 1 Centro di Oncobiologia Sperimentale, University of Palermo, 90146 Palermo, Italy; lucadicar@gmail.com (G.D.C.); rosi82.m@libero.it (R.M.); patrizia.cancemi@unipa.it (P.C.); nadianinfa.albanese@gmail.com (N.N.A.); salvatoreminafra@gmail.com (S.M.); 2 Dipartimento di Scienze e Tecnologie Biologiche Chimiche e Farmaceutiche, University of Palermo, 90128 Palermo, Italy; 3 La Maddalena Hospital, 90146 Palermo, Italy; roz@lamaddalenanet.it

**Keywords:** breast cancer, surgical tissues, gel-based proteomics, mass spectrometry, protein clustering

## Abstract

The present investigation has been conducted on one hundred tissue fragments of breast cancer, collected and immediately cryopreserved following the surgical resection. The specimens were selected from patients with invasive ductal carcinoma of the breast, the most frequent and potentially aggressive type of mammary cancer, with the objective to increase the knowledge of breast cancer molecular markers potentially useful for clinical applications. The proteomic screening; by 2D-IPG and mass spectrometry; allowed us to identify two main classes of protein clusters: proteins expressed ubiquitously at high levels in all patients; and proteins expressed sporadically among the same patients. Within the group of ubiquitous proteins, glycolytic enzymes and proteins with anti-apoptotic activity were predominant. Among the sporadic ones, proteins involved in cell motility, molecular chaperones and proteins involved in the detoxification appeared prevalent. The data of the present study indicates that the primary tumor growth is reasonably supported by concurrent events: the inhibition of apoptosis and stimulation of cellular proliferation, and the increased expression of glycolytic enzymes with multiple functions. The second phase of the evolution of the tumor can be prematurely scheduled by the occasional presence of proteins involved in cell motility and in the defenses of the oxidative stress. We suggest that this approach on large-scale 2D-IPG proteomics of breast cancer is currently a valid tool that offers the opportunity to evaluate on the same assay the presence and recurrence of individual proteins, their isoforms and short forms, to be proposed as prognostic indicators and susceptibility to metastasis in patients operated on for invasive ductal carcinoma of the breast.

## 1. Introduction

Breast cancer is still at the top of the statistics for the incidence among women worldwide but, fortunately, the percentage of survival has significantly increased in all the western countries [1]. A recent retrospective study has reported that the five-year relative survival for the localized breast cancer is 99%, while it drops to 84% when the cancer cells have spread to local lymph nodes, and to 26% when the cancer cells have invaded distant anatomic region and form metastasis [2,3]. These results demonstrate that the primary prevention and early surgical removal save the lives of nearly all women with breast cancer in the early stages.

The challenge now is to predict in time the propensity of the primary tumor to form metastases and to be able to tackle or block them in time.

Unfortunately, at present, there are not sufficient and accurate prognostic markers that can predict the metastatic ability of tumor cells at the time of surgical resection, although many researchers are seriously engaged in this challenge [4,5].

A major complication is that breast cancer is not a single disease: indeed clinical and pathological evidence suggests the existence of several groups and subgroups of breast cancer, within the two major histological types of ductal and lobular origin, each of them often displaying different biological and clinical histories. In the majority of cases patients belonging to distinct subtypes respond differently to the treatments and have dissimilar long-term survival rates [6].

Despite the histopathological differences among mammary tumors, there are similarities on the conversion of a localized primary breast tumor to the invasive form, a process known as tumor progression. A basic step involved in the tumor progression is the stepwise disruption of cell-cell and cell-matrix contacts, followed by the loss of the polarized morphology, typical of the stationary epithelial phenotype. Concurrently with the cell detachment from the tissue boundaries, the neoplastic cells pass through the basal lamina and acquire a mesenchymal motile phenotype, while the cell-surface becomes unstable displaying a ruffled appearance, with a tendency to release vesicles of a different nature [7,8]. This phenomenon known as the epithelial/mesenchymal transition (EMT) is supported by a set of genes inducing neoplastic cells to gain migratory and invasive properties towards the surrounding and distant tissues [9,10].

The present work is a retrospective study of 100 surgical fragments obtained and cryopreserved over the past years from patients operated on for invasive ductal carcinoma, which is the most frequent and aggressive form of breast cancer.

The objective of this work was the identification of potential tumor markers for breast cancer by applying the proteomic technique based on two-dimensional separation of the proteins followed by their identification by mass spectrometry. This procedure permits to obtain simultaneous information on the same assay, related to the occurrence of hundreds of proteins, their p*I* and MW coordinates, their isoforms and short forms potentially useful as prognostic or predictive biomarkers. To our knowledge, other important large-scale proteomic approaches are mainly bases on collective technical separation, performed by multicenter investigation teams [11,12].

## 2. Materials and Methods

### 2.1. Clinical Specimens

Tissue aliquots of invasive ductal carcinoma of the breast (IDC) were obtained and immediately cryopreserved, following surgical interventions during the years 2003–2007 at the “La Maddalena” Hospital of Palermo, and intended for elimination after the completion of the histopathological examination.

Research was carried out in compliance with the Helsinki Declaration with the patients’ written consent. The patients of this study did not receive any therapeutic treatment before to surgery.

### 2.2. Sample Preparations

Aliquots of the frozen tissues (ranging from 0.5 to 1.00 g) were washed several times with PBS and homogenized by a Polytron device in an ice bath. The homogenizing medium was: RIPA buffer (50 mM Tris pH 7.5, 0.1% Nonidet P-40, 0.1% deoxycholate, 150 mM NaCl, 4 mM EDTA), containing a mixture of protease and phosphatase inhibitors (0.01% aprotinin, 10 mM sodium pyrophosphate, 2 mM sodium orthovanadate, 1 mM PMSF) [13]. The extraction was conducted overnight at 4 °C. The cellular lysate was centrifuged to remove debris, and the supernatant was dialyzed against ultrapure distilled water, lyophilized and stored at −80 °C.

### 2.3. Two-Dimensional Gel Electrophoresis

The protein extracts were dissolved in a buffer composed by 4% CHAPS (3-[(3-Cholamidopropyl) dimethylammonio]-1-propanesulfonate) (Sigma-Aldrich, St. Louis, MO, USA), 40 mM Trizma base (Sigma-Aldrich, USA), 65 mM DTE (1,4-Dithioerythritol) (Sigma-Aldrich, USA) and a trace of bromophenol blue in 8 M urea. Protein concentration was determined by the Bradford assay [14].

Protein samples (45 µg for the analytical gels, or 1.5 mg for preparative gels) were rehydrated in a solution containing 8 M urea, 2% CHAPS, 10 mM DTE and 0.5% carrier ampholytes (Resolyte 3.5–10; Amersham, Little Chalfont, UK), and applied to the strips for isoelectrofocusing (IEF) (18 cm long, pH range 3.0–10, Bio-Rad, Hercules, CA, USA). After the IEF the strips were incubated in a solution composed by 50 mM Tris-HCl pH 6.8, 6 M urea, 0.5% SDS, 30% Glycerol, 130 mM DTE and 135 mM Iodoacetamide (Sigma-Aldrich).

The focused proteins were then separated on 9–16% linear gradient polyacrylamide gels (SDS-PAGE) with a constant current of 20 mA/gel at 10 °C. The protein spots were revealed by ammoniacal silver staining [15].

Silver-stained gels were analyzed with ImageMaster 2D Platinum software, Version 5.0 (Amersham, Little Chalfont, UK) with the support of the ExPaSy molecular biology server.

### 2.4. Protein Identification

The protein identity was searched by peptide mass fingerprinting using the Voyager DE-PRO (AbSciex, Framingham, MA, USA) MALDI-TOF mass spectrometer as described [16]. In-gel digestion of the protein spots was performed with sequencing-grade trypsin (Promega, Madison, WI, USA), and the peptides were re-dissolved in 0.1% trifluoroacetic acid (TFA) and spotted in HCCA (R-cyano-4-hydroxycinnamic acid) matrix (Sigma-Aldrich). The mass spectra were recorded in the 500–5000 Da range, using a minimum of 150 shots of laser per spectrum. Internal calibration was performed using trypsin autolysis fragments at *m*/*z* 842.5100, 1045.5642, and 2211.1046 Da. Peptide mass fingerprinting was compared to the theoretical masses from the Swiss-Prot databases using the Mascot algorithms (http://www.matrixscience.com/). Search parameters were: 50 ppm of mass tolerance, carbamidomethylation of cysteine residues, oxidation of methionine residues and one missed enzymatic cleavage for trypsin. A minimum of four peptide mass hits was required for a match.

**Figure 1:**
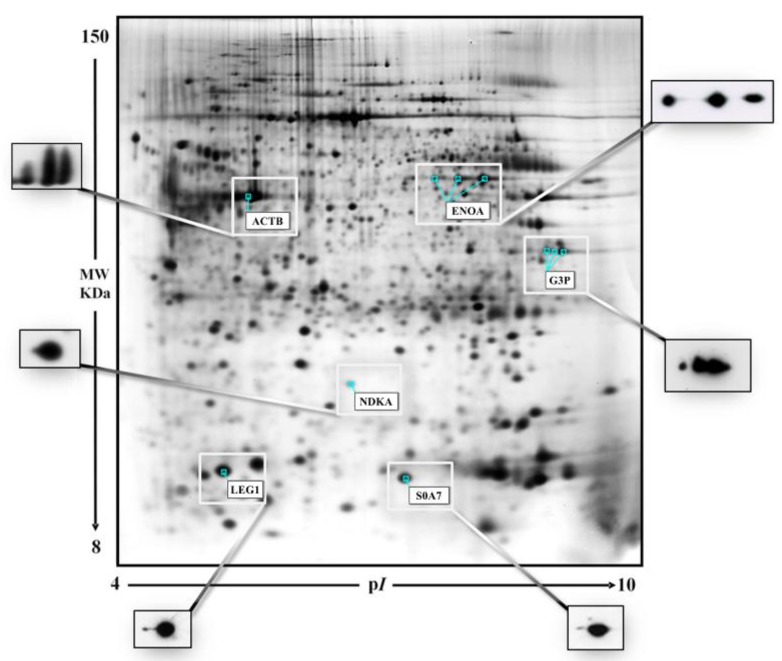
A representative gel image of a breast cancer tissue. The graphic white boxes highlight the protein spots used as “anchors” for the gel alignment by the algorithms of the Image Master software. The side panels show the 2D-Western blot validations of the selected spots with the proper antibodies (anti-actin, anti-GADPH, anti-enolase, anti-LGALS1, anti-nm23-H1 and anti-psoriasin).

For the qualitative/comparative analysis the proteomic maps were matched by the algorithms of the software Melanie utilizing immunologically validated anchors in the reference map (Figure 1).

For the comparative-quantitative analysis of the protein expression, the ratio between the average intensities of individual protein spots in tumor over the non-tumoral tissues was calculated. Protein classification was performed according to the major databases [17,18]. The proteins discussed in the text are indicated with full name, abbreviated name and gene name (in brackets), according to the ExPaSy molecular biology server.

### 2.5. Western Blot

For protein immune detection, the 2D-gels were electrotransferred onto nitrocellulose membrane (HyBond ECL, Amersham) and stained with Ponceau S (Sigma Aldrich) as previously described [19]. The membranes were then probed with the following monoclonal antibodies (mAb): anti-actin (clone JLA20) mouse mAb (1:1000; Merck KGaA); anti-GADPH (clone 0411) mouse mAb (1:1000; Santa Cruz Biotechnology, Dallas, TX, USA, SCBT); anti-enolase (clone A5) mouse mAb (1:1000; Santa Cruz Biotechnology, SCBT); anti-LGALS1 (clone 1E8-1B2) mouse mAb (1:1000; Novus Biologicals, Littleton, CO, USA); anti-nm23-H1 (clone 37.6) mouse mAb (1:1000; Santa Cruz Biotechnology, SCBT); and anti-psoriasin (clone 47C1068) mouse mAb (1:1000; Santa Cruz Biotechnology, SCBT. Following incubation with goat anti-mouse IgG-HRP (1:5000, Santa Cruz Biotechnology, SCBT), the reaction was revealed by the ECL detection system, using high performance films (Hyperfilm ECL, Amersham).

## 3. Results

Firstly, we wanted to compare the proteomic expression between the tumor tissues of breast cancer with healthy counterparts of the same patient.

**Figure 2:**
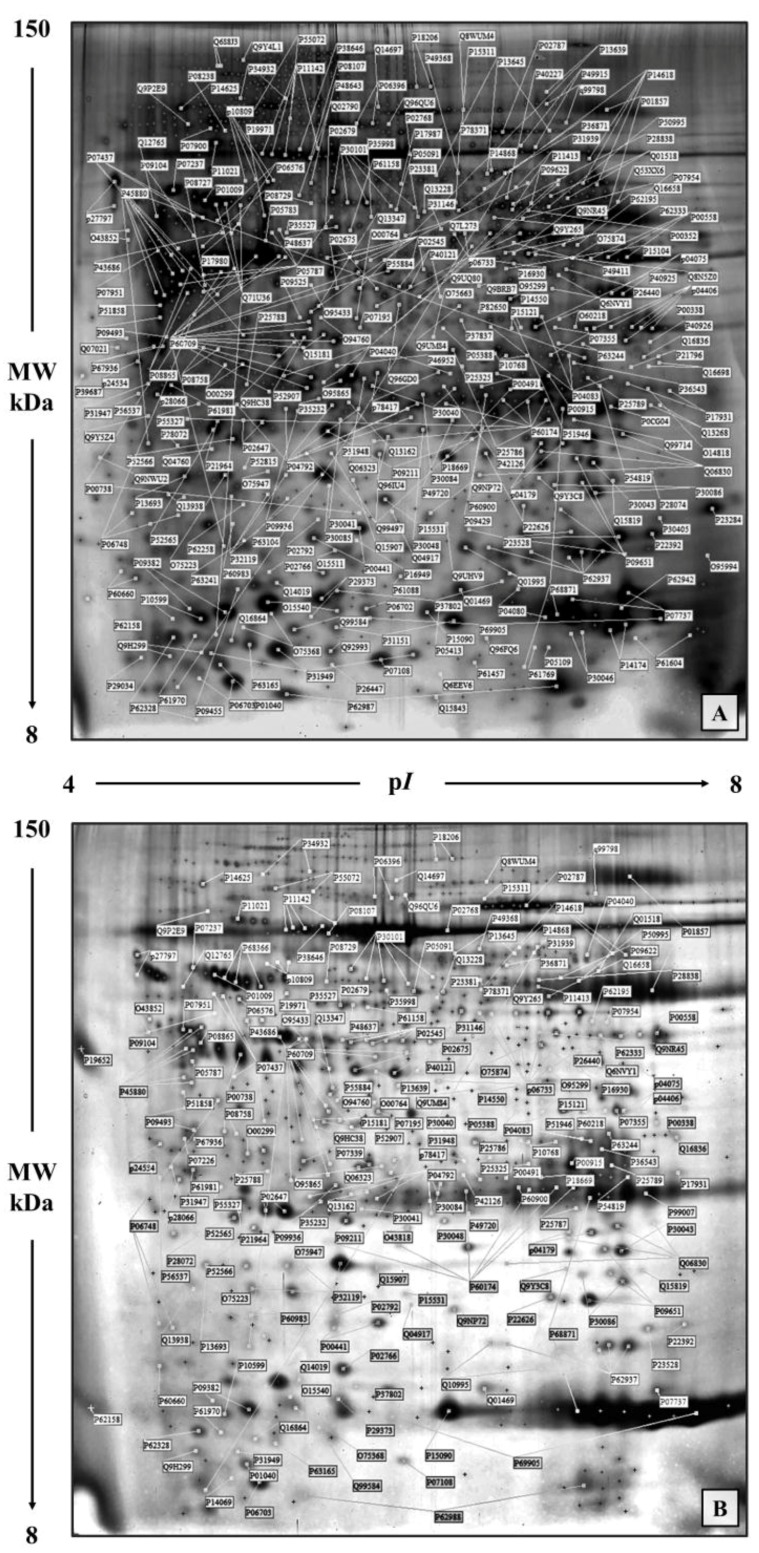
Representative proteomic maps of a breast cancer tissue (**A**) and its non-tumoral adjacent tissue (**B**). Protein spots of known identity are labelled with the access number of the Swiss-Prot/TrEMBL database. Different isoforms of the same protein are jointly labelled.

This analysis was previously conducted on a group of 13 patients with infiltrating ductal carcinoma (IDC) of the breast [20]. In Figure 2A,B, a prototype of twin maps, tumoral and non-tumoral updated to an increased number of spots (453 spots corresponding to 271 genes) is shown. For the qualitative/comparative analysis the maps were matched by the algorithms of the ImageMaster 2D Platinum software, utilizing immunologically validated anchors in the reference map, as shown in Figure 1. The quantification of the matching profiles was performed after normalization of the spot intensity values to the actin levels, as cellularity index [21] to correct for the different content of extracellular proteins in the tissue fragments.

**Figure 3:**
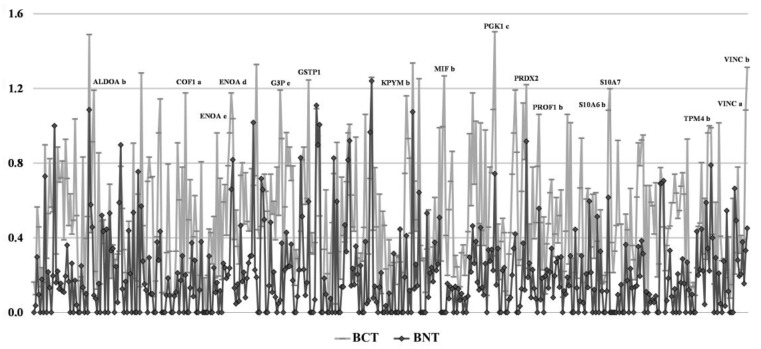
Global densitometric profiles of the proteomic maps shown in Figure 2, illustrating the relative differences in the actin-normalized intensity values of protein spots from tumoral (BCT, grey line) and non-tumoral adjacent tissue (BNT, black line).

The graph in Figure 3 shows the layout of the two comparative densitometric profiles, where the differential pattern between tumoral and non-tumoral tissues is evident. Applying a cutoff value of 2 between the tumoral and non-tumoral tissues, more than 50% of the detected proteins showed higher expression levels in the tumors, while the remaining appeared unchanged and only a minority displayed a lower expression. Among the over-expressed proteins were: some glycolytic enzymes, different isoforms of S100 proteins, several proteins of the cell motility, and some proteins with anti-apoptotic activity.

Supported by these results, we extended the investigation to an increasing number of breast cancer cases, focusing the research on the invasive ductal carcinoma (IDC) which is one of the most frequent and aggressive form of breast cancer. Here we report a retrospective proteomic analysis performed on 100 surgical fragments of patients operated on during the time lapse of the years 2003–2007.

These analyzes allowed us to verify the heterogeneous proteomic expression among patients. In particular, some proteins proved to be expressed in all of the tested cases (albeit at differential intensity) while other proteins showed an occasional or sporadic presence among the patients [22]. We considered ubiquitous proteins those present in at least 90% of patients and sporadic the remainder, meaning absent the spots missing perceptible signal in the matched maps.

Table S1 (supplementary data) shows the catalogue of proteins collectively identified by 2D-IPG followed by mass spectrometry of the individual spots. Different isoforms of the same protein were labelled by alphabetical letters starting with *a*.

The differential patterns of the proteins expressed in the 100 maps is represented in the diagrams reported in the supplementary Figure S1, where in the *x-axis* are the patients ordered with progressive numbers and in the *y* the protein names. The white boxes represent the presence and the grey ones the absence of individual protein spots. As a whole, the ubiquitous proteins were 63% of identified total proteins while the sporadic ones were 37% of the total identified spots.

### 3.1. Ubiquitous Proteins

Among the ubiquitously over-expressed proteins, worthy of note was the detection of several isoforms of key glycolytic enzymes, several proteins with anti-apoptotic activity, members of the S100 protein family and several members of the proteasome complex. Concerning the S100 proteins, eight family members were ubiquitously expressed among the patients and seven were sporadic.

Moreover, a generalized expression of a typical mesenchymal marker, the cytoskeletal vimentin, was found in contraposition to the statistical decrease of cytokeratins in the patients.

**Figure 4:**
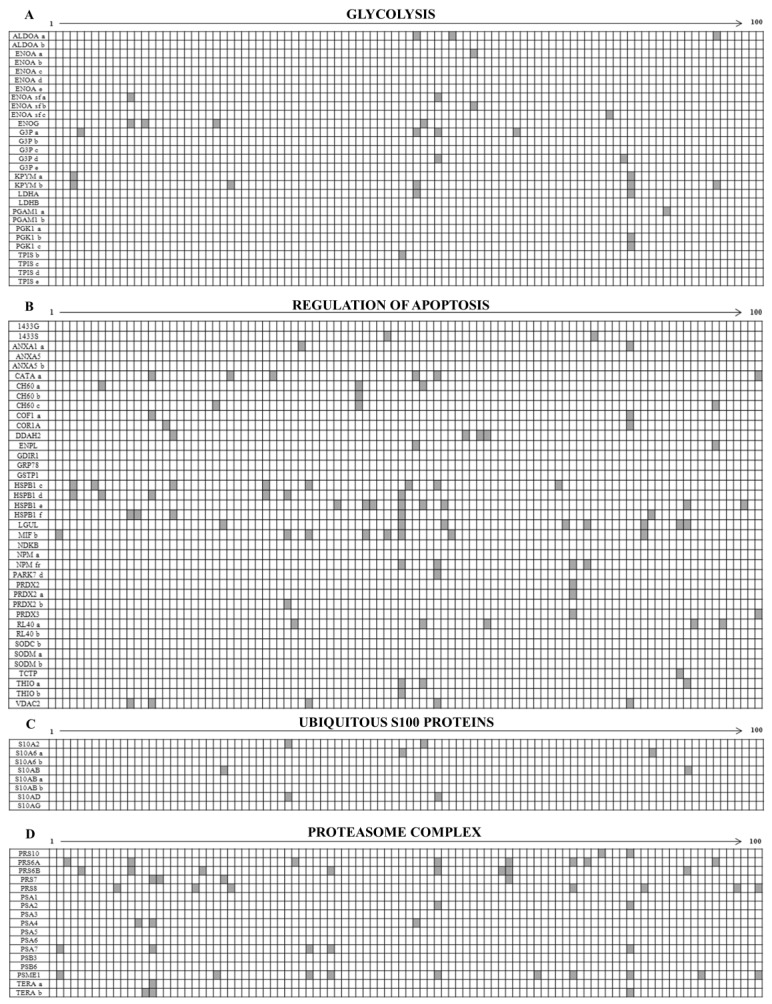
Diagrams illustrating the occurrence of the ubiquitous proteins (ordinate axis) among the 100 studied patients (abscissa axis). Proteins are indicated according to the Swiss-Prot/TrEMBL database. Isoforms are reported with alphabetical letters. The white boxes indicate the presence of a given protein (or isoform) and the grey boxes its absence in the corresponding patient map. The ubiquitous proteins are sorted in the following classes: (**A**) Glycolysis; (**B**) Regulation of Apoptosis; (**C**) S100 Proteins with ubiquitous expression and (**D**) Proteasome complex.

The diagram in Figure 4A shows the occurrence of glycolytic enzymes, including isoforms (29 members); the diagram 4B represents the occurrence of a “transverse” class of heterogeneous proteins (39 members), each performing peculiar functions, which share the activity of apoptosis regulation, performed through diverse pathways; the diagram 4C shows the occurrence of the ubiquitous S100 proteins (8 members), and the diagram 4D represents proteins of the proteasome complex (17 members).

**Figure 5:**
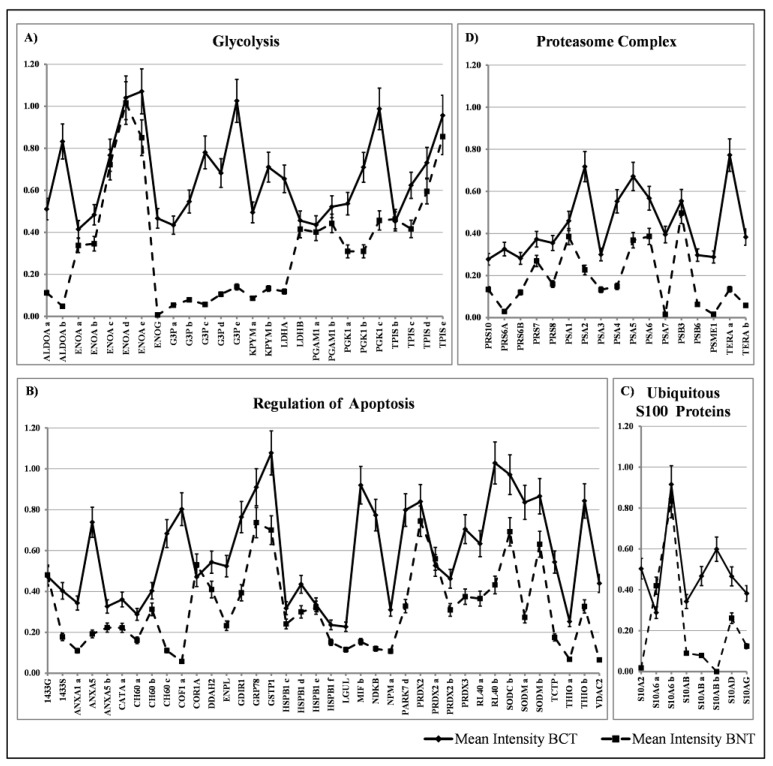
Comparative expression of the normalized average intensity levels between the ubiquitous proteins in the breast cancer tissues (BCT, continuous line) and in the non-tumoral tissues (BNT, dashed line). The ubiquitous proteins are sorted in the following groups: (**A**) Glycolysis; (**B**) Regulation of Apoptosis; (**C**) S100 Proteins with ubiquitous expression and (**D**) Proteasome complex. The bars indicate the standard deviation.

To assess the levels of the mean expression of these groups of ubiquitous proteins, the average values of the individual proteins and their isoforms were plotted against the media of 10 selected healthy tissues as a reference. The quantification of the expression levels of the individual protein spots was evaluated through the intensity values normalized to the actin content in each map [21] and reported in Figure 5A–D. As can be observed in the diagrams, some of the glycolytic enzymes did not show differential expression levels between normal and breast cancer tissues, namely the ENOA forms, the two isoforms of PGAM1 and the 5 isoforms of TPIS. Conversely, the other detected enzymes of the glycolytic pathway displayed a significant increase with respect to the corresponding normal ones, namely: the two isoforms of ALDOA, the 5 isoforms of G3P, the 4 isoforms of KPYM and the 3 forms of the PGK1. Moreover, the high expression of the ENOG, usually absent in the non-tumoral tissues, was observed.

Concerning the group of regulators of apoptosis, all members showed significant increase in the tumoral tissues, with a maximum peak for cofilin (about 14 folds).

In the group of S100 proteins, the only member that did not show any variation between normal tissues and the tumors, was the S100A6 with its two isoforms; all the other members displayed a significant increase in cancer.

Finally, the group of proteasome complex subunits (17 members) showed a significant increase with respect to the normal counterpart.

The remaining proteins, not included in the above reported classes, were recognized by the databases as belonging to the following functional classes: protein folding, tricarboxylic acids cycle, protein biosynthesis and exosomes.

### 3.2. Sporadic Proteins

**Figure 6:**
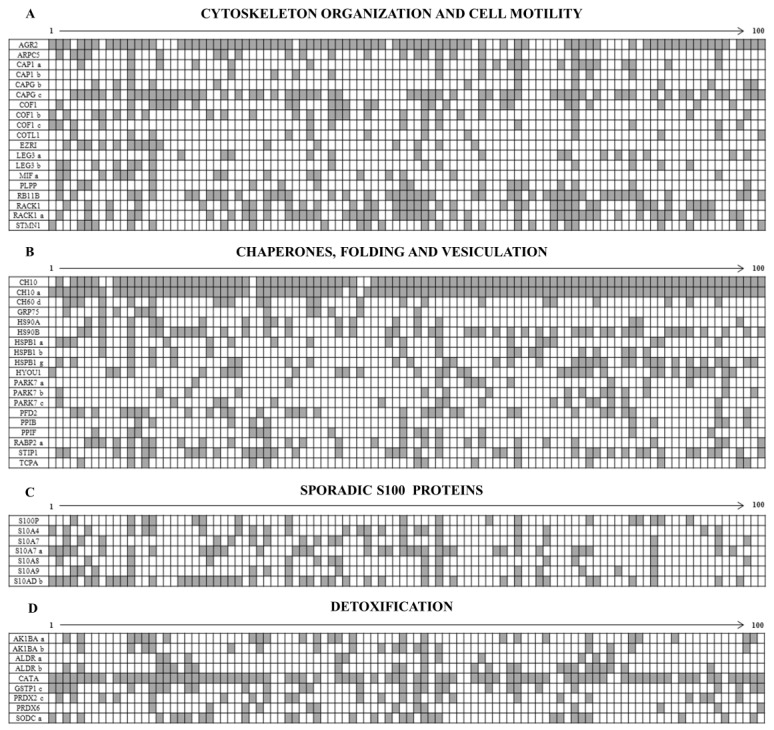
Qualitative data analysis illustrating the occurrence of the sporadic proteins (ordinate) among patients, indicated by 1 to 100 (abscissa), as reported in Figure 4. The sporadic proteins are sorted in the following classes: (**A**) Cytoskeleton Organization and Cell Motility; (**B**) Chaperones, Folding and Vesiculation; (**C**) S100 Proteins with sporadic expression and (**D**) Detoxification.

**Figure 7:**
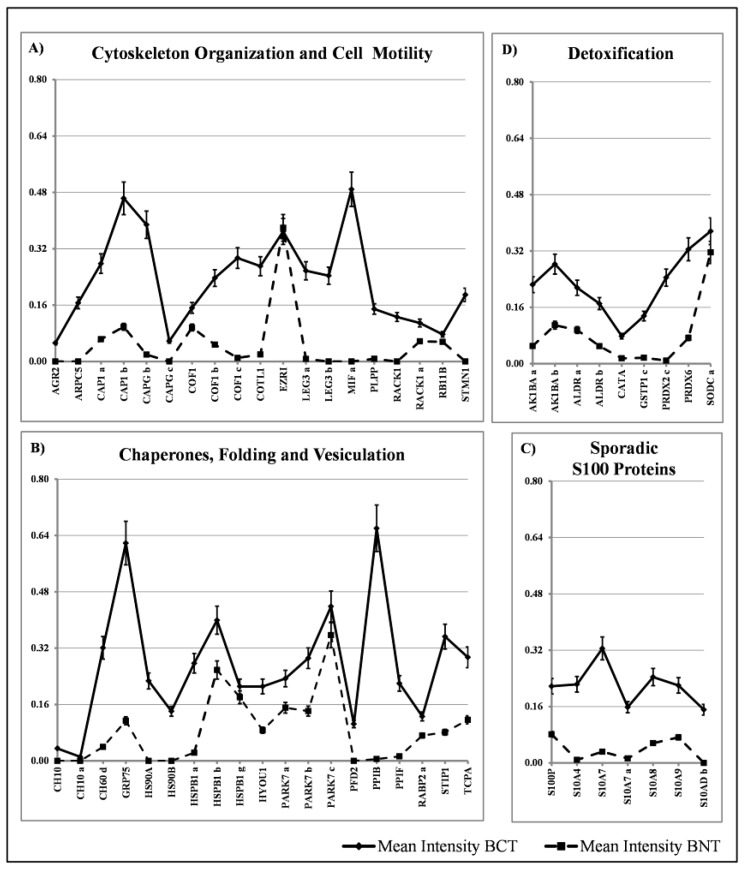
Comparative expression of the normalized average intensity levels between the sporadic proteins in the breast cancer tissues (BCT, continuous line) and in the non-tumoral tissues (BNT, dashed line). The sporadic proteins are sorted in the following classes: (**A**) Cytoskeleton Organization and Cell Motility; (**B**) Chaperones, Folding and Vesiculation; (**C**) S100 Proteins with sporadic expression and (**D**) Detoxification. The bars indicate the standard deviation.

Within the group of proteins with sporadic occurrence among the tissues, we have identified four major classes: proteins involved in cytoskeleton organization and cell motility (19 members), chaperon and stress response proteins (19 members) and detoxification proteins (9 members) and S100 proteins (7 members). The diagrams showing their distribution are reported in Figure 6A–D. The comparative quantification of the expression levels of these proteins are reported in Figure 7.

The remaining proteins, not included in above classes, were recognized by the databases as belonging to the following functional classes: cell adhesion, nucleotide binding, mitochondrial proteins, intracellular transport and exosomes.

### 3.3. Intermediate Filament Proteins

**Figure 8:**
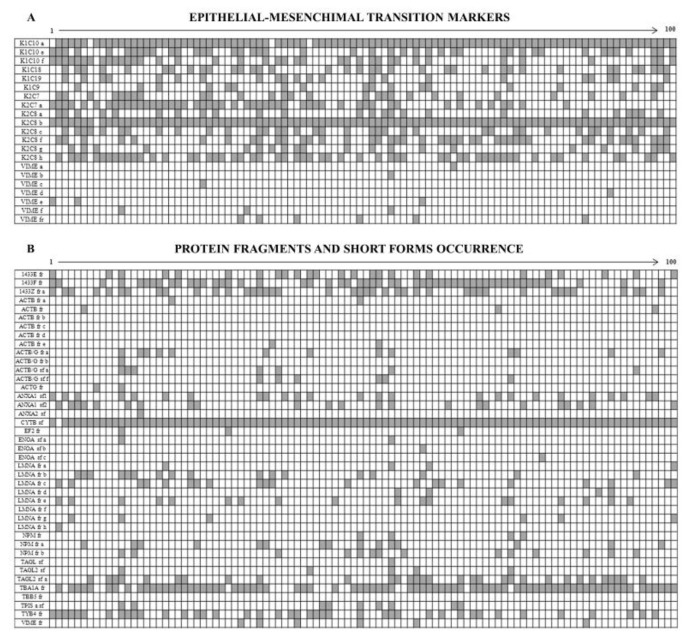
Qualitative diagrams illustrating the occurrence of: (**A**) Epithelial-Mesenchimal transition markers and (**B**) Protein fragments-Short forms. Proteins are listed in the ordinate axis and tumor patients (listed by 1 to 100) in abscissa.

Figure 8A shows the diagram of the opposite occurrence of 14 cytokeratin members and 6 vimentin isoforms. Namely the cytokeratins, all sporadically expressed, were: three isoforms of K1C10, two isoforms of K2C7, 6 isoforms of K2C8, K1C18, K1C19 and K1C9. By contrast all the identified vimentin isoforms were ubiquitously expressed in all the patients, so testifying that all tumors display a clear epithelium/mesenchyme transition.

### 3.4. The Enigma of Protein Fragments and Short Forms

Among the 453 identified proteins we found 41 incomplete protein forms (9%), which were subdivided into “short forms” (13 over 41) and “fragments” (28 over 41), on the basis of their molecular weight differences in relation to the canonical forms (Figure 8B).

We defined “short forms” polypeptides whose molecular mass is lower by up to 30% of their corresponding canonical form, while we classified as “fragments” the proteins whose mass was more than 30% lower than that of the canonical form.

Both the fragments and the short forms displayed a reproducible two-dimensional electrophoretic migration pattern in all the analyzed samples, having in each proteomic profile the same isoelectric point and molecular mass. The majority of these incomplete forms (63.4%) was ubiquitously expressed on the analyzed breast cancer samples, whereas the remaining occurrence was sporadic.

## 4. Discussion

One of the current challenges for the treatment and prevention of cancer relates to the identification of suitable markers to be used to prevent or monitor the progress of the disease.

The 2D-based proteomic strategy allows to identify hundreds of proteins on the same assay and to compare a large number of cases at the same time, using computerized systems. The present investigation has been focused on 100 surgical tissue fragments of invasive ductal carcinoma of the breast collected and cryo-preserved over the years at the La Maddalena Hospital of Palermo.

The protein identification was done through the two-dimensional gel electrophoresis followed by the peptide mass fingerprinting. This method allows attributing at the same time the molecular coordinates of a protein (predicted MW and p*I*), its isoforms and the mass-fingerprinting of its peptides, generated by in situ cleavages of the spot of interest.

At present we have unequivocally identified 453 protein spots reported in a reference proteomic map referred to the 100 selected patients. To evaluate the occurrence and frequency of individual proteins and their isoforms, we have built an extensive Cartesian-style diagram where the patients (indicated by the increasing numbers) were reported in the axis of abscissas and the protein abbreviated names in the axis of ordinates.

Proteins detected in at least 90% of the patients, and therefore designed as ubiquitous, represented 63% of the total, while the remaining 37% displayed a sporadic presence within the patients.

### 4.1. Ubiquitous Proteins

Among the ubiquitously overexpressed proteins, about 9% belongs to the category of glycolytic enzymes and their isoforms, 13.3% belongs to the transverse group of proteins with regulative activity on apoptosis, 2.8% to the S100 proteins, 5.6% to the Proteasome complex and the others are scattered among the various classes of proteins with metabolic or structural functions.

#### 4.1.1. The Overexpressed Glycolytic Enzymes

It is known since the times of Otto Warburg [23], who first described the phenomenon, that cancer cells exhibit a transition from the aerobic metabolism to the anaerobic glycolysis. This event, formerly interpreted as a consequence of a hypoxic environment within the tumor mass, appears plausible, but not exclusive to justify the increased protein expression of several key enzymes of the glycolysis, also under normoxic conditions “in vitro” [24], and in mammospheres [25]. Indeed, increased levels of gene expression of several glycolytic enzymes have also been detected in several cancer types [26].

Our present results showed that in a statistical average of 100 cases of invasive ductal carcinoma an increase of some key glycolytic enzymes occurred, namely: ALDOA, G3P KPYM, PGK1, and LDHA, while no relevant increase was observed for other glycolytic enzymes (i.e., ENOA, PGAM1, TPIS).

Several investigations during the past years have, however, revealed numerous other non-glycolytic functions carried out by different enzymes among the canonical 10 of the glycolytic pathway.

*Fructose-bisphosphate aldolase A, ALDOA (ALDOA)*. Literature data shows that ALDOA is over-expressed in many types of cancers, including hepatocellular carcinoma [27], squamous cell lung cancer [28,29], osteosarcoma [30], colorectal cancer [31]. The recognized non-glycolytic functions of ALDOA include: participation to signal transduction [32], vesicle trafficking [33], cell motility [34] and epithelial-to-mesenchymal transition [35]. In addition, ALDOA may be phosphorylated by Akt or Erk 2 kinases and then it can move into the nucleus, where it may be involved in the regulation of transcription of genes implied in cell cycle progression [36] and the DNA protection [37].

The ubiquitous overexpression of ALDOA in all of the 100 patients studied supports the relevance of this increased expression in driving or sustaining elevated proliferation rhythm in cancer.

*Glyceraldehyde-3-phosphate dehydrogenase, G3P (GAPDH)*. The GAPDH gene has been classically used as a housekeeping gene, but in more recent years it has been shown to be over-expressed in many tumors including breast cancer and to be correlated with a poor prognosis [38] and an increased drug resistance [39]. In particular, it has been reported that GAPDH overexpression is associated with cell proliferation via its effects following direct or indirect (through binding with the protein SET) interactions with cyclin B-cdk1 [40]. These effects concern mainly an acceleration of cell cycle progression and an increased number of mitosis. Moreover, it has been demonstrated that when the G3P binds to its substrate, the glyceraldehyde-3-phosphate, the small GTPase Rheb is released and it works as a positive regulator of mTORC1, an essential pathway regulating many cellular responses to growth factors [41]. These additional functions of the enzyme depend in particular on the dislocation from its native cytoplasmic location. Indeed, it can migrate to the nucleus or to the mitochondria where it may perform additional functions beyond the glycolytic pathway [42,43], this also by post-translational modifications induced, for example, by exposure to various stresses [44]. Moreover, the deregulation of glycolytic enzymes in cancer may cause cascading consequences with adverse outcomes for patients. Among the most remarkable targets are membrane trafficking, microtubule assembling, phosphotransferase activity, binding of nucleic acids [45]. For these reasons, G3P is presently included in the possible therapeutic targets [46].

The capability of G3P to promote cell proliferation, together with its elevated and ubiquitous expression in our breast cancer tissues, strongly supports an oncogenic role for this protein.

*Pyruvate kinase, KPYM (PKM)*. It has been demonstrated that this enzymes, besides its role in the glycolytic pathway, interacts and cooperates with Oct-4, a gene which encodes a transcription factor performing a significant role in sustaining the pluripotent state of embryonic stem cells and preventing the expression of differentiation genes [47].

It is worth noting that in our 100 cases of tumors, two of the four isoforms were ubiquitously over-expressed.

*L-lactate dehydrogenase A chain, LDHA (LDHA)*. It has been reported in the literature that this enzyme, involved in step 1 of the glycolytic subpathway promotes cancer cell invasion, anoikis resistance, and tumor metastasis, via HER2 and Src pathways [48,49]. It has also been shown that an experimentally increased expression of 14-3-3ζ in human mammary epithelial cells up-regulates LDHA expression, elevates glycolytic activity, and promotes early transformation through the MEK-ERK-CREB axis [50]. Present data supports these mentioned literature records.

*Phosphoglycerate kinase 1, PGK1 (PGK1)*. Many types of cancers, including those of the breast [51], the colon [52], the liver [53] and others, have been shown to exhibit an increased expression of PGK1. Indeed, it has been demonstrated that, in addition to its role as a glycolytic enzyme, PGK1 may act as a promoter of cancer progression and induce chemoresistance for some drugs [51,52]. Moreover, this protein may be secreted by tumor cells, in spite of the absence of the appropriate signal sequence, and participate in the “angiogenic switch” by reducing disulfide bonds in the serine protease, plasmin [54,55]. The ubiquitous overexpression of PGK1 in our 100 tumor samples supports the hypothesis of the important role played by this protein in tumorigenesis.

Finally, it is worth mentioning that the other key enzyme of the glycolytic pathway, the ENOA, is highly expressed both in normal and tumoral tissues. On the contrary, the ENOG, which is known to be specific for nervous tissues, is almost absent in the non-tumoral tissues and ubiquitously expressed at very high levels, in the tumor tissues. Its role in breast cancer has not yet been clarified [56].

#### 4.1.2. Regulators of Apoptosis

Apoptosis is a genetically programmed process which brings cells to suicide following different injuries. In normal conditions of tissue renewal and during development, there is a controlled balance between cell division and cell death. This equilibrium is lost during carcinogenesis, where cell proliferation prevails over cell death. There are two major apoptotic pathways, extrinsic and intrinsic, involving some different proteins, the majority of them also performing several different cellular roles [57]. Recently a paradox of function has been attributed to the apoptosis in cancer, postulating a pro-tumoral function of the apoptotic process [58].

The group of proteins that we classified under the name of “regulators of apoptosis” is a “transverse” group of proteins since they perform several distinct functions, but they share the apoptosis regulation activity, documented in the gene ontology databases [59]. Some of these proteins also perform important roles in the complex scenario of the progression of various cancers.

With the exclusion of COF1, which shows ambivalent roles, all the other members were classified as anti-apoptotic proteins by the databases.

The proteins included in this group are indicated below.

*The 14-3-3 multi-gene family*. The multigenic protein family 14-3-3 includes several members that can generate homo- or heterodimers [60]. In mammals seven genes (β, ε, η, γ, τ, ζ and σ) have been identified so far, all of them acting as key regulators of several intracellular signaling pathways. These include cell cycle regulation, apoptosis, control of metabolism and gene transcription [61], and maintenance of epithelial cell polarity [62]. The mechanisms of action of the 14-3-3 proteins are varied according to their specific functions in tissues: indeed they may activate or stabilize some proteins and inactivate others; in many cases, they perform a role of scaffold molecules [63]. In our proteomic maps, we have recognized 4 out of 7 members of the family (β, γ, ζ and σ), having different occurrence in cancer tissues. Among these, the 14-3-3 γ and 14-3-3 σ were found ubiquitously expressed in all patients, while the other isoforms have been found sporadically present among the patients.

Several studies in the field of oncology have demonstrated that the tumoral transformation is associated with the onset of antiapoptotic mechanisms. In this respect, a major anti-apoptotic and pro-proliferative role of the 14-3-3 proteins, within a complex network of protein-protein interactions in cancer, has been highlighted [64,65,66].

The occurrence of the gamma form in 100% of our breast cancer cases and 98% of the sigma form underlines the important role of these isoforms in raising the anti-apoptotic activity during the primary tumor growth, so confirming and strengthening the involvement of the 14-3-3 proteins in the molecular processes critical for tumor progression.

*The Annexins*. Annexins consist of 13 members of Ca^2+^ and phospholipid binding family of peripheral membrane proteins. Mostly, they can bind the phospholipid of the membranes in a Ca^2+^ dependent manner and perform subsequent activities related to membrane trafficking, signal transduction, and exocytosis [67]. In our proteomics study, we have found a ubiquitous and over-expressed presence of ANX1A and of two isoforms of ANXA5 which are known to play an anti-apoptotic role and are thought to be involved at different levels in the tumor development, progression and invasivity [68,69,70]. Therefore both of them are regarded as potential tumor markers [71], a hypothesys shared by this study.

*Catalase, CATA (CAT)*. The catalase is an antioxidant enzyme which plays the first line of defense against free radicals, removing hydrogen peroxide from cells. It is known to exert an anti-apoptotic role in several tumors [72]. In our tissues, we detected two isoforms of CATA, one of which appeared ubiquitously expressed, but with a modest average increase with respect to the normal tissues.

*Cofilin, COF1 (CFL1)*. The COF1 is a protein mainly involved in the actin cytoskeleton organization during the normal progress through mitosis and cytokinesis. It is also required for the maintenance of cell morphology and the cell motility [73]. Concurrently, it is involved in the regulation of apoptosis. However, in this context, it appears to have ambivalent roles, since in many cases it showed to play pro-apoptotic activity and in others anti-apoptotic [74,75,76]. In our samples we have identified four cofilin isoforms, one of these was ubiquitous, displaying a very higher expression level (14×) on the non-tumoral tissues. New literature evidence associates the high expression levels of cofilin and its dephosphorylated form, with poor prognosis in breast cancer patients [77].

*The ubiquitous heat-shock proteins* (HSPs). The heat-shock proteins, formerly discovered as proteins of response to heat-stress injury, represent a large protein family mainly involved in protein folding and playing significant roles in cellular proliferation, differentiation, and protection of cells from stress [78]. According to their molecular size the HSPs have been classified into six major families (HSP100, HSP90, HSP70, HSP60, HSP40, and small heat shock proteins sHSPs). Some of them perform their functions in both the cytosolic and the nuclear compartments (i.e., HSP90), others in the endoplasmic reticulum (i.e., GRP94) or other subcellular compartments (i.e., the mitochondrial HSP70) [79].

*HSPB1 (HSPB1) and CH60/HSP60 (HSPD1)*. It has been reported that the HSPs display elevated expression levels in cancer, where they may perform anti-apoptotic activities both spontaneous and generated by therapy [80]. In particular, the high expression of HSPB1 has been associated with poor prognosis in several carcinomas and osteosarcomas [81], while CH60, with annexin-2, is considered a potential biomarker for subtypes of lung carcinoma [82]. A result of the interest of our study, which deserves further investigation, was the detection of seven isoforms of HSBP1, four ubiquitous and three sporadic, and of four isoforms, three ubiquitous and one sporadic of the CH60. One of the ubiquitous forms of CH60, that appeared the closest to the expected values for the primary gene product, showed a high average increase (6 folds) with respect to the non-tumoral tissues.

*78 kDa glucose-regulated protein*, *GRP78* (HSPA5). This protein, also known as Binding immunoglobulin Protein (BiP), or Heat Shock 70 k, is a member of the heat shock protein 70 (HSP70) family. Its primary function is that of molecular chaperone within the lumen of the endoplasmic reticulum, where it binds newly synthesized proteins as they are translocated into the ER. Its anti-apoptotic role has also been reported [83]. In addition, it may regulate the TGF-β pathway, through the molecular interaction with its ligand Crypto, an oncofetal GPI-anchored/secreted signaling protein, which plays a key role as a stem cell regulator [84]. For these reasons, Cripto/GRP78 complex represents a possible therapeutic strategy for the treatment of human cancer [85]. Interestingly, this protein has been found ubiquitously present in all of our patients and sporadically in the non-tumoral tissues.

*Endoplasmin, ENPL/GRP-94 (HSP90B1)*. The endoplasmin, paralogue of the cytosolic heat shock protein 90 (HSP90), is a molecular chaperone for the processing and transport of secreted proteins; it has been shown to be a ligand for multiple receptors including Toll-like receptors, Wnt and integrins [86]. More recent preclinical studies have also revealed that GRP94 expression is closely related to advanced stage and poor survival of patients in a variety of cancers [87]. This protein was ubiquitously expressed in our tissues where it showed a moderate increase versus the non-tumoral tissues.

*Lactoylglutathione lyase LGUL (GLO1)*. This enzyme catalyzes the conversion of hemimercaptal, formed from methylglyoxal and glutathione to S-lactoylglutathione. It is also involved in the regulation of transcriptional activity of NF-kappa-B induced by TNF. Interestingly, it appears to be required for normal osteoclastogenesis and therefore involved in the complex mechanism of bone metastasis [88]. In our tumor tissues, LGUL showed a moderate fold increase on the non-tumoral tissues.

*Macrophage Migration Inhibitory Factor, MIF* (MIF). This factor is involved in several biological activities which ultimately may support cancer progression [89]. The expression of this protein stimulates the production of cytokines, chemokines, and growth factors, as well as angiogenic factors, that favor the tumor growth also potentiating its aggressiveness and metastatic spreading [90,91]. The overexpression of MIF has been demonstrated in breast cancer cells, in which, through the interaction with HSP90 and CXCR-4, MIF induces resistance to the apoptosis and stimulates the proliferation via AKT pathway [91]. In our tissues, we found two isoforms of MIF, one of which ubiquitous and highly overexpressed (6×).

*Nucleoside diphosphate kinase B, NDKB (NME2)*. The gene coding for this protein was the first anti-metastatic gene to be discovered [92] and correlated with good prognosis in multiple tumor types [93]. It plays a major role in the synthesis of nucleoside triphosphates other than ATP. Moreover, it regulates negatively Rho activity by interacting with AKAP13/LBC, and may act as a transcriptional activator of the MYC gene [94]. In our samples, the NDKB showed a significant fold increase (6.4×) versus the non-tumoral tissue.

*Nucleophosmin, NPM (NPM1)*. The NPM1 plays critical roles in many cellular processes, including protein chaperoning, cell proliferation and apoptosis. In consideration of its multifunctional potentiality NPM1 can play roles both as a proto-oncogene and as a tumor suppressor [95,96]. In our tumor tissues, we observed its moderate increase on the non-tumoral tissues.

*Parkinson disease protein 7, PARK7/DJ1 (PARK7)*. This protein plays a central function as deglycase in several cytoplasmic pathways. It is also involved in protecting cells against oxidative stresses and apoptosis, acting as oxidative stress sensor and redox-sensitive chaperone. It is mutated in several neurodegenerative disorders, most notably Parkinson’s disease [97]. In the context of cancer, it has been suggested that it promotes cell survival by enhancing AKT phosphorylation and thus the inhibition of PTEN function [98,99]. It has been reported that in patients with lung, ovarian and oesophageal cancers, high expression of PARK7 predicts a poor outcome [100]. Moreover, high expression of PARK7 in breast cancer potentiates HER3 signaling and therefore may serve as a target for molecular therapies [101]. In our tissue samples, we have identified four isoforms one of which was ubiquitous and moderately over-expressed on the non-tumoral tissues.

*Superoxide dismutase (Mn) mitochondrial, SODM (SOD2)*. The SODM is an enzyme which destroys superoxide anion radicals normally produced as metabolic waste, toxic to biological systems. It has been demonstrated that SOD2 exerts an anti-apoptotic effects attributed to its ability to generate H_2_O_2_ [102]. This enzyme appeared ubiquitously increased in all of our tumoral tissues (3×).

*Translationally-controlled tumor protein, TCTP (TPT1)*. The classical roles of this protein are the calcium binding and microtubule stabilization. In addition, it has been found engaged in the epithelial to mesenchymal transition and in the promotion of cell migration, invasion, and metastasis [103,104]. In our tumoral tissues, we found a single form of it, increased of 3× with respect to the non-tumoral tissues.

*Thioredoxin, THIO (TXN)*. The thioredoxin is a small protein playing pivotal roles in redox homeostasis and cell survival, and it is usually highly expressed in many cancers [105]. Indeed, in response to reactive oxygen species (ROS) associated with the tumors, thioredoxin and DJ-1 are upregulated to limit or reverse cell damages [106,107]. In our samples, we found two close isoforms of thioredoxin increased respectively of 3.7 and 2.5 folds in tumors.

*Voltage-dependent anion-selective channel protein 2, VDAC2 (VDAC2)*. This protein is a member of the VDAC family and shares structural homology with the other VDAC isoforms (VDAC1 and VDAC3), which are involved in the regulation of metabolite diffusion across the mitochondrial outer membrane. It has been stated that the overexpression of VDAC2 prevents BAK activation and inhibits the mitochondrial apoptotic pathway [108]. The high increase (6.7×) of this anti-apoptotic protein in all the patients studied is a further indication of the importance of the anti-apoptotic systems to ensure the unchallenged growth of cancer cells.

No relevant differences in the average of expression levels between tumoral and non-tumoral tissues were observed for the other components of the group: i.e., COR1A, DDAH2, GDIR1, GSTP1, PRDX2, PRDX3, RL40.

#### 4.1.3. Ubiquitous S100 Proteins 

The S100 proteins are members of a multigenic family of calcium-binding proteins of the EF-hand type, encoded by 21 genes in human [109]. Members of the S100 family are differentially distributed in tissues [110] where they perform a great variety of functions. They may act intracellularly or may be released into the microenvironment and exert extracellular regulatory effects [111]. Intracellular functions include calcium homeostasis, regulation of phosphorylation, regulation of gene expression, cytoskeleton dynamics and cell motility. Their extracellular activities are fulfilled in a cytokine-like manner through the *receptor for advanced glycation end products* (RAGE) [112]. Some of the secreted S100 proteins may exert chemotactic [113] and antibacterial actions [114].

Their frequent altered expression has been correlated with various aspects of the tumor growth and progression [115]. In our breast cancer tissues, we recognized 15 members, including isoforms, corresponding to 10 genes, eight of them (corresponding to 5 genes) were ubiquitous.

*S10A2 (S100A2)*. Among the ubiquitous S100 protein members, the S100A2 is the one showing a higher index of fold increase (about 30 times) on the average of non-tumor tissues. This significant increase agrees with the role attributed in the literature to this protein [115]. However, concerning its role in the apoptosis, literature data shows an often contradictory role of S100A2 which in some cases works as a tumor suppressor [116], and in others, as a tumor promoter [117].

*S10A6 (S100A6)*. This protein was found expressed at high levels in both tumor and non-tumoral tissues, confirming the key role and leadership attributed to this protein, as calcium sensor and modulator, in sustaining many physiological processes, among which the reorganization of the actin cytoskeleton and in cell motility [118].

*S10AB (S100-A11)*. This is a calcium-binding and cadherin-binding protein also involved in cell-cell adhesion. In our tissues we detected three isoforms of it, all of them expressed at high levels in tumors and nearly absent in the normal tissues. Literature data reports that the increased expression of S100A11 contributes to the growth of certain tumors, due its activity of negative regulation of cell proliferation [119,120].

*S10AD (S100A13)*. S10AD has been proposed to play roles in the invasiveness of lung cancer cells [121] and angiogenesis of human melanoma [122]. In our tissues, we found two isoforms of it, one of which ubiquitous, showing a moderate fold increase, and the second, more basic, expressed sporadically.

*S10AG (S100A16)*. This member of the S100 protein family has been reported to be involved and overexpressed in several pathological affections, including cancer, but with different and often contradictory roles [123,124,125]. In our tumor tissues, we found a single form of S10AG with a fairly high increase (3×) versus the normal tissues.

#### 4.1.4. Proteasome Subunits

The high proliferation rate in cancer has destabilizing effects on many cytoplasmic proteins. There are at least two systems to counteract these disadvantages: the system of degradation, of which the ubiquitin-proteasome is the leader, and the chaperone system consisting of various stress-induced proteins (HSPs). A proper capability to degrade the misfolded proteins is crucial for cell survival, while the impairment of this function contributes to the aging and neurodegeneration. More recently, a paradoxical function has been proposed for the proteasome system as being a promoter of cell survival and tumor progression [126].

In our tissue maps, we detected the ubiquitous presence of 15 proteasomal subunits coded by 15 genes. The majority of them showed a higher average expression on the non-tumoral tissues, with peaks for PRS6A (11×), PSME1 (18×), and PSA7 (26×).

### 4.2. Epithelium-Mesenchymal Transition (EMT) Markers

A result of considerable interest was the identification of the concurrent expression of key elements of the epithelial-mesenchymal transition in the tumor samples: indeed eight protein spots corresponding to vimentin and its fragments were ubiquitously present in all samples, while 14 CK forms, isoforms and fragments, were highly sporadic among the tissues.

The phenomenon of epithelium-mesenchyme transition has been much studied in recent years [9,10,127] and represents one of the key features in the transition from a stationary to a motile cellular phenotype during tumor progression. To our knowledge, this is the first evidence of a collective expression pattern at the proteomic level of the EMT pattern in breast cancer and may be of great utility for more accurate and personalized histo-pathological diagnoses.

### 4.3. Sporadic Proteins 

We classified as “sporadic” the proteins that occur in less than 90% of the patients studied. These represent 37% of the spots identified in proteomic maps. They were divided into four main classes with the support of the database DAVID [17]: cell motility, heat shock, detoxification, sporadic S100.

These proteins were expressed at low or very low levels in the non-tumoral tissues.

#### 4.3.1. Cell Motility Proteins 

Motility and migration are distinctive activities of embryonic cells. Adult epithelial cells are characterized by polarized, adherent and stationary cell sheets, owing to the constraints of cell-cell and cell-matrix adhesions: these are destroyed during the primary tumor growth, due to the activities of the matrix metalloproteinases that are over-expressed in the site of primary tumor and, in many cases, released into the circulation [128].

The loss of polarity and stationarity produces cascading effects, including primarily the rearrangement of the cytoskeleton and the reactivation of cell motility. Thus, the reorganization of the actin cytoskeleton plays a central role in the migration of potentially metastatic cells [129].

**Figure 9:**
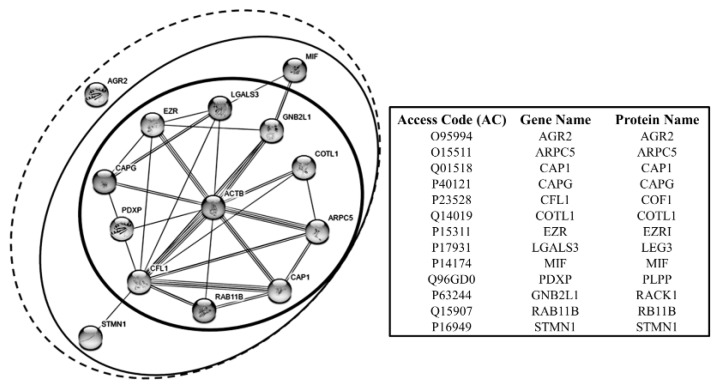
Interactome graph of the interrelationships between cell motility proteins. Each circle encloses the proteins according to the interaction level with ACTB that represents the focal node of this network. Graphs constructed by the support of STRING—Known and Predicted Protein-Protein Interactions [17].

It is interesting to note that most of the proteins of this group belong to the category of the actin binding proteins. In Figure 9 the interactome of these proteins is represented, showing that some of them exhibit a direct link with actin (at the center of the network), and others hold indirect actin links through the primaries.

This group includes the proteins commented below.

*Actin-related protein 2/3 complex subunit 5, ARPC5 (ARPC5)*. ARPC5 is a component of the ARP2/3 complex, with cofilin, RAB118 and others, which plays a key role in the remodeling of the actin cytoskeleton, to direct many forms of cellular motility, including vesicle traffic [130]. As shown by the interactome in Figure 9, ARPC5 interacts directly with ACTB, COTL1, CAP1, COF1, RAB11B. Given its strategic role in supporting the motile capability of cells, the role of ARPC5 in the tumor progression is well sustained [131]. This discovery enables the ARP complex to be a viable candidate for a molecular targeted therapy against invasion and metastasis [132]. Within our collection of tumor samples, the ARPC5 expression recurred in 78% of cases at the high expression level, while it proved to be absent, or at the detection limit, in the normal tissues.

*Adenylyl cyclase-associated protein 1, CAP1 (CAP1)*. The actin-binding CAP1 protein regulates directly actin filament dynamics, therefore participating in important cellular processes, including the establishment of cell polarity. Its overexpression in some types of tumors has been related to the increase of the metastatic potential of cancer cells [133]. In our tumor tissues, we observed the sporadic presence of two isoforms of CAP1 with a high increase versus the non-tumoral tissues (4.3× and 4.7×. respectively).

*Gelsolin-Like Capping Protein, CAPG (CAPG)*. CAPG is a calcium-sensitive protein which blocks reversibly the barbed ends of actin filaments. Its overexpression in cancer has been correlated with the mechanisms of cell migration and invasiveness and more recently has been associated with breast cancer progression and bone metastases formation [134]. In our samples, we detected two isoforms, one of which with a very high increase (19.7×), versus the non-tumoral tissues expressing this protein.

*Cofilin1, COF1 (CFL1)*. In addition to its function in the control of apoptosis, cofilin1 plays a central role in the dynamics of actin-cytoskeleton remodeling. As shown by the interactome in Figure 9 COF1 interacts directly with ACTB, ARPC5, and CAP1. Indeed, it is involved in the turnover of the actin branches, so promoting actin filaments treadmilling and participating in the formation of cell protrusions related to the motile phenotype [135]. This attitude has been proven to be enhanced in malignant cancer cells [136,137]. As already reported, in our tissue we have found four COF1 isoforms, on of which ubiquitous and three sporadic. Interestingly, one of the sporadic isoform, when present, displayed one of the highest average increase (29×) compared to the non-tumoral tissues. The role of COF1 in the regulation of apoptosis has been already discussed.

*Coactosin, COTL1 (COTL1)*. Coactosin interacts with actin in a calcium-dependent manner, downstream of the Rac pathway, so promoting the actin polymerization during the formation cell protrusions [138]. Recently coactosin expression levels have been associated with the metastatic potential of lung cancer cell lines [139]. In our samples, it showed a very high expression levels (13.6×) with respect to the non-tumoral tissues. As shown in the interactome, COTL1 interacts directly with ACTB, ARPC5, and COF1.

*Ezrin, EZRI (EZR*). EZRI is a member of the ERM family, together with radixin and moesin and others, is involved in signal transduction, protein trafficking, cell proliferation, migration and in the establishment and maintenance of the epithelial cell polarity [140]. The EZRI overexpression is closely related to the breast cancer malignant phenotype, in which the EZRI role seems to be dual: the participation to the cell protrusion dynamics and to the ERBB2 receptor signaling [141]. It is of interest to note, that EZRI is among the proteins of the motility that show the highest expression level both in tumoral and non-tumoral tissues. The interactome in Figure 9, shows that EZRI may interact directly with ACTB, LEG3, COF1 and CAPG.

*Galectin, LEG3 (LGALS3)*. LEG3 is a galactose-specific lectin associated to the cell membrane and involved in several processes of the membrane coating and trafficking by interacting with other surface proteins, including the ANXA2 [142]. Intracellularly LEG3 participates to the cytoskeleton organization and cell motility by interacting with ACTB, COF1, EZRI, MIF and CAPG (Figure 9). Extracellularly, it interacts with a variety of cell surface glycoproteins, i.e., growth factor receptors, integrins and members of the Notch family, and other extracellular matrix molecules [143]. In cancer, LEG3 seems to participate to the membrane dynamics of cell migration and in the escaping of the T cells-mediated immune-response [141]. By its overexpression and of its functions in the breast cancer cells, LEG3 has been recently candidate as a biomarker for the triple-negative breast cancer progression [144,145]. In our tumor samples LEG3, when present, showed a very high expression level, while it was absent in the normal tissues.

*Macrophage Migration Inhibitory Factor, MIF (MIF)*. As already reported MIF is an inflammatory cytokine involved in a great number of cellular pathways, including the control of apoptosis [90]. During the invasive progression of breast cancer, MIF appears involved in the phenomena of trans-endothelial cells migration, related to the intra- and extra-vasation processes [91].

As previously indicated, in our tissues we detected two isoforms of MIF, one ubiquitous and the second sporadic with a high level of expression and absent in the normal tissues. For its proteomic co-expression with other proteins associated with cell motility, its attribution to this group of proteins is plausible, but this does not exclude its belonging also the anti-apoptotic group. MIF enters in the interactome pathway through the interaction with LEG3.

*Pyridoxal phosphate phosphatase, PLPP (PDXP)*. PLPP is a serine phosphatase, having among its target cofilin. Indeed, the PLPP activity is considered crucial for the regulation of the actin-cytoskeleton rearrangement mediated by the cofilin [146]. In our maps, it shows a very high expression level (20×) compared to the non-tumoral tissues.

*Receptor of activated protein C kinase, RACK1 (RACK1)*. RACK1 is involved in the recruitment and assembly of a variety of signaling molecules, thus playing a role in many cellular processes. Indeed, it has been reported that RACK1 may play a role in the promotion of breast cancer cell migration, by binding to and activating RHOA [147]. Interestingly, RACK1 appears absent in the non-tumoral tissues, while when present in the tumors it is expressed at high levels of intensity. In Figure 9, its interaction with ACTB, MIF, EZRI and COF1 is shown.

*Ras-related protein Rab-11A, RB11B (RAB11B)*. This protein belongs to the Rho family small GTPases and, as the other Rho proteins, it is crucial for the machinery of the vesicular-based intracellular trafficking and for actin reorganization related to the cell motility processes [131]. A recent study demonstrated a functional relationship between RB11B and the moesin, a member of the ERM family (Ezrin, Radixin and Moesin), in the mechanisms which coordinate the collective cell migration [148]. In our maps RB11B, when present, displayed a relatively low increase compared with the normal tissues.

*Stathmin, STMN1 (STMN1)*. Stathmin is a phosphoprotein mainly involved in the regulation of the microtubule system in a dual way: by preventing assembly and by promoting disassembly of microtubules. Some evidence associates the overexpression of the STMN1 to the cancer progression. Indeed, it has been reported that the MEK-mediated phosphorylation of STMN1 is a key process of cancer cell migration [149]. Concerning the actin cytoskeleton, stathmin shows to interact with the COF1 (Figure 9). Interestingly, in our samples stathmin was found highly expressed in some tumors and absent in the non-tumoral tissues.

*Anterior gradient protein 2 homolog, AGR2 (AGR2)*. This protein may be secreted from cells, and in the ECM context, it can potentiate the tumorigenic effects on responding cells [150]. AGR2 expression was shown to be significantly increased in HER2 positive breast tumors [151]. High levels of AGR2 were also associated with cell dissemination of pancreatic carcinoma [152] and with poor prognosis of lung cancer [153]. Worth of note, this protein appears highly represented in the tumor tissues (when present) and almost absent in the normal ones.

#### 4.3.2. Sporadic Heat Shock Proteins 

As already reported, a notable presence of HSP proteins in the tumor tissues was observed. Some of them were expressed ubiquitously in the tissues and already commented in relation to their key roles in the control of apoptosis. Other HSPs were found sporadically in our tumor tissues and almost absent in the normal ones. Many of them have been proposed by several authors as putative biological markers for tumor progression [80,81,154].

The sporadic heat shock proteins are indicated below.

*CH10 (HSPE1) and CH60 (HSPD1).* These two chaperonines form a complex mainly involved in the import of mitochondrial proteins and in the macromolecular assembly. Their involvement in the tumor progression is still not fully elucidated. The expression level of CH10 in our tumoral and non-tumoral tissues is under the threshold value of the other Heat Shock Proteins. Concerning CH60, as already reported, we found four isoforms, three ubiquitous and one sporadic, the latter was expressed at high level in the tumor tissues, while it was very sporadic in the normal ones and, when present, it was moderately expressed.

*GRP75 (HSPA9)*. GRP75, also known as “mortalin”, is a mitochondrial chaperone belonging to the subgroup of the glucose-related proteins. GRP75 is positively involved in the carcinogenetic process, due to its ability to prevent the transcriptional activation and the pro-apoptotic functions of the p53. It has been documented that its over-expression correlates with the increase of breast carcinoma cell malignancy and with poor prognosis for breast cancer patients [155,156]. In our tumor tissues, when present, it reached high expression values compared to the non-tumoral tissues (5.4×).

*Hypoxia up-regulated 1 protein, HYOU1 (HYOU1)*. HYOU1 belongs to the heat shock protein 70 (HSP70) family. Its primary function is closely related to the cellular responses triggered by oxygen deprivation; it also plays an important role as a molecular chaperone. HYOU1 results over-expressed in many tumors, where it appears associated with tumor invasion and with the inhibition of the drug-induced apoptosis [157]. This protein, when present, displayed a moderate fold increase on the normal ones.

*HS90A (HSP90AA1) and HS90B (HSP90AB1)*. HSP90s, displaying cytoplasmic and nuclear localization, play key roles in the protein homeostasis by regulating the folding of many proteins involved in the control of cell growth, signaling and in the pathogenesis of several human diseases, especially cancer [158]. It has been demonstrated that the nuclear HSP90 regulates the activity of many transcription factors and the activity of RNA polymerase II [159]. In our tissue samples, we found a marked increase in the expression levels of two members of the HSP90 family, namely HS90A and HS90B, on the non-tumoral tissues.

*Parkinson disease protein 7, PARK7/DJ1 (PARK7)*. As already reported in the previous paragraph, in our tumor tissues this protein is expressed (at moderate intensity) with four isoforms having similar MW and different p*I*. One of them was ubiquitously expressed and already discussed, the others were found sporadically expressed in the tumors, but also in some of the normal tissues.

*Cyclophilin B, PPIB (PPIB)*. PPIB is a 21-kDa protein having isomerase activity. Its overexpression in cancer seems to contribute to the neoplastic transformation and progression, through its ability to regulate the levels of several hormone receptors [160] and to influence the expression of key genes for the control of cell proliferation and cell motility [161]. In our samples, we found a striking abundance of this sporadic protein, which is also occasionally present in the non-tumoral tissues at very low levels.

*Cyclophilin D, PPIF (PPIF)*. PPIF is a mitochondrial peptidyl-prolyl isomerase. It acts as a regulator of the mitochondrial permeability by participating to the control of the pore dynamics. Although its role in cancer is still unclear, recent studies reported that PPIF up-regulation seems to play a role in the tumor progression, through the Paf1/MEK/ERK pathway [162,163]. When present, in our tumor tissues, this protein was expressed at very high levels.

*Prefoldin 2, PFD2 (PFDN2)*. PFD2 is a subunit of the prefoldin complex, localized in the cytoplasm and also into the nucleus and crucial for the folding of actin and tubulin [164]. Its overexpression has been associated with poor prognosis in the bladder carcinoma [165]. Interestingly, this protein is expressed at high levels in the tumor tissues and absent in the non-tumors.

*Cellular retinoic acid-binding protein 2, RABP2 (CRABP2)*. RABP2 is a retinoic acid binding protein whose principal role is the delivery of retinoic acid to its nuclear receptor, so modulating the expression of a large number of genes involved in the control of cell proliferation. The second function of RABP2 is the stabilization of the RNA-binding protein HuR and the strengthening of its interactions with target mRNAs [166]. RABP2 expression is associated with the growth suppression in a large number of carcinomas playing a putative anti-oncogenic action [167]. A moderate intensity of it has been detected in our tissue samples.

*Stress-induced phosphoprotein 1, STIP1 (STIP1)*. STIP1 is a co-chaperone of HSP-70 and -90 and participates in the JAK2/STAT3 pathway by stabilizing JAK2 protein. The downstream signaling of JAK2/STAT3 pathway activates several mechanisms responsible for the progression of ovarian and endometrial carcinomas [168]. When present, this protein is expressed at low levels in the normal tissues and at moderate levels in the tumoral tissues.

*TCP-1-alpha, TCPA (TCP1)*. TCPA is a molecular chaperone involved in the folding of actin, tubulin and a large number of proteins (i.e., Cyclin E, Cyclin B and p21^ras^) with a role in cell proliferation and neoplastic progression. Its expression is regulated by the signaling downstream tot the PI3K pathway. According to recent data, the TCPA appears necessary for the in vitro and in vivo survival and growth of breast cancer cells [169,170]. A moderate increased level of it was detected in the tumors.

#### 4.3.3. Sporadic S100 Proteins 

As already mentioned, in our breast cancer tissues we recognized 15 members of S100 family proteins, seven of which corresponding to six genes, were sporadic as mentioned below. All of them, when present, showed remarkably increased levels on the normal tissues.

*S100P (S100P)*. S100P is known to function as calcium sensor and to interact with several regulators of cytoskeleton rearrangement, thus contributing to several cellular physiological processes.

It stimulates cell proliferation and survival via activation of the receptor RAGE [171,172] in an autocrine manner.

*S10A4 (S100A4)*. The S100A4 protein is known to be secreted by tumor and stromal cells and to support tumorigenesis by stimulating angiogenesis. Indeed, it has been demonstrated that S100A4 synergizes with vascular endothelial growth factor (VEGF), via the RAGE receptor, in promoting endothelial cell migration and MMP-9 and MMP-13 gene expression. For this reason, S100A4 protein is today included in the catalog of the putative therapeutic target [173].

*S100A7 (S100A7)*. Within the sporadic forms of S100 proteins, the S100A7 was one of the most prominent, also for the high level of relative concentration reached in many cases. Its possible role in breast cancer has been discussed in a previous work by our group [174] and other authors [175,176,177].

*S10A8 (S100A8), S10A9 (S100A9)*. These two members of the S100 protein family are known to play also significant roles in the extracellular fluids during various inflammatory conditions, such as rheumatoid arthritis, cystic fibrosis and abscesses [178].

*S100AD (S100-A13)*. As previously reported, in our tumor tissues the S100AD displays two isoforms, the first is ubiquitous and the second, more basic, was sporadic in tumor and absent in the normal tissues. The functional role of this S100 protein has been already discussed [121,122].

#### 4.3.4. Detoxification Proteins

The detoxification processes are crucial for the cellular homeostasis in response to the accumulation of metabolic waste, derived from intracellular pathways or from extracellular responses to external stimuli, which may interfere with the basic biological functions of the cells. In the neoplastic cells, this accumulation reaches high levels due to the increased anaerobic metabolism.

The antioxidant enzyme system is very complex, being composed of some vitamins, primary and secondary antioxidant enzymes and thioredoxin systems [179].

In our tumor tissues, we identified the sporadic expression of 9 detox proteins (encoded by seven genes), namely: SODC, CATA, PRDX2, PRDX6, GSTP1, ALDR and AK1BA. All of them showed higher expression levels, with a peak of 27.5 for the PRDX2, as compared with the normal tissues.

It is noteworthy that ALDR, AK1BA and GTSP1 have been associated with cancer progression. ALDR, AK1BA are enzymes involved in activation and detoxification of carcinogenic polycyclic aromatic hydrocarbons, a key process for the malignancy in hepatocellular cancer [180]. GSTP1 is involved in the detoxification functions, however, its silencing appears closely related to the neoplastic transformation. This evidence suggests a possible role for GSTP1 as a prognostic marker [181].

## 5. Conclusions

In conclusion, the data obtained on a large scale proteomics platform made it possible to give an overview on the expression of proteins which appear to be protagonists of a tumor phenotype.

The proteomic analysis of proteins expressed ubiquitously in all patients compared to the sporadic proteins suggests that the phase of the primary tumor growth must be sustained and supported by protein clusters capable of ensuring a sustained proliferation not counteracted by normal apoptotic processes that govern the normal tissue renewal.

Even greater interest was stimulated by the discovery of overexpression to high levels of glycolytic enzymes, the role of which, the so-called Warburg effect, has already been known since the time of its discoverer. In fact, two of the key enzymes of the glycolysis, the G3P and the ALDOA, are recognized as proteins with important oncogenic regulatory functions [182,183]: the first, once nitrosylated enters into the nucleus and activates gene transcription pathways that support cell proliferation, and the other that in addition to its role in glycolysis is able to promote epithelial-mesenchymal conversion.

Consequently, primary tumor growth can be ensured by two concordant pathways: the inhibition of apoptosis and the stimulation of proliferation by the deregulated activity of housekeeping proteins, including some key enzymes of the glycolysis. We believe that the second phase of the evolution of the tumor, namely invasion and metastasis, is supported by activities of genes and proteins involved in a later stage of growth, in particular: proteins involved in cell motility, several S100 proteins and proteins of the detox machinery, which were sporadic between patients, index probably of a susceptibility to metastasis in patients who were carriers of these over-expressed proteins.

Future perspective is, therefore, large-scale clinical and molecular correlations with selected cluster of gene/proteins and follow-up of newly enrolled patients.

## Supplementary Materials

Supplementary materials are available online at http://www.mdpi.com/2227-7382/5/3/15/s1. Figure S1. Panel summarizing the occurrence of the 453 proteins identified in the studied 100 breast cancer tissues, Table S1: Catalogue of the 453 proteins identified in the studied 100 breast cancer tissues.

## References

[1] Hashim D., Boffetta P., La Vecchia C., Rota M., Bertuccio P., Malvezzi M., Negri E. (2016). The global decrease in cancer mortality: Trends and disparities. Ann. Oncol..

[2] Susan G. Komen. http://ww5.komen.org/BreastCancer/ChancesForSurvivalBasedOnCancerStage.html.

[3] Howlader N., Noone A.M., Krapcho M., Miller D., Bishop K., Altekruse S.F., Kosary C.L., Yu M., Ruhl J., Tatalovich Z. (2016). SEER Cancer Statistics Review 1975–2013.

[4] Weigel M.T., Dowsett M. (2010). Current and emerging biomarkers in breast cancer: Prognosis and prediction. Endocr. Relat. Cancer..

[5] Wang Y. (2015). Development of cancer diagnostics from biomarkers to clinical tests. Transl. Cancer Res..

[6] Breast Cancer Fund. http://www.breastcancerfund.org/clear-science/biology-of-breast-cancer/breast-cancer-subtypes.

[7] Pucci-Minafra I., Karamanos N.K. (2012). Extracellular matrix in breast cancer: Permissive and restrictive influences emanating from the stroma. Extracellular Matrix: Pathobiology and Signalling.

[8] Palazzolo G., Albanese N.N., Di Cara G., Gygax D., Vittorelli M.L., Pucci-Minafra I. (2012). Proteomic analysis of exosome-like vesicles derived from breast cancer cells. Anticancer Res..

[9] Minafra L., Bravatà V., Forte G.I., Cammarata F.P., Gilardi M.C., Messa C. (2014). Gene expression profiling of epithelial-mesenchymal transition in primary breast cancer cell culture. Anticancer Res..

[10] Minafra L., Norata R., Bravatà V., Viola M., Lupo C., Gelfi C., Messa C. (2012). Unmasking epithelial-mesenchymal transition in a breast cancer primary culture: A study report. BMC Res. Notes.

[11] Waldemarson S., Kurbasic E., Krogh M., Cifani P., Berggård T., Borg Å., James P. (2016). Proteomic analysis of breast tumors confirms the mRNA intrinsic molecular subtypes using different classifiers: A large-scale analysis of fresh frozen tissue samples. Breast Cancer Res..

[12] Mertins P., Mani D.R., Ruggles K.V., Gillette M.A., Clauser K.R., Wang P., Wang X., Qiao J.W., Cao S., Petralia F. (2016). Proteogenomics connects somatic mutations to signalling in breast cancer. Nature.

[13] Di Cara G., Marengo G., Albanese N.N., Marabeti M.R., Musso R., Cancemi P., Pucci-Minafra I. (2013). Proteomic Profiling of Trastuzumab (herceptin®)-sensitive and resistant SKBR-3 Breast Cancer Cells. Anticancer Res..

[14] Bradford M.M. (1976). A rapid and sensitive method for the quantitation of microgram quantities of protein utilizing the principle of protein-dye binding. Anal Biochem..

[15] Pucci-Minafra I., Fontana S., Cancemi P., Basiricò L., Caricato S., Minafra S. (2002). A contribution to breast cancer cell proteomics: detection of new sequences. Proteomics.

[16] Pucci Minafra I., Di Cara G., Musso R., Peri G., Valentino B., D’Arienzo M., Martini D., Raspanti M., Minafra S. (2016). Proteomic profiling of In Vitro bone-conditioned skbr3 breast cancer cells. J. Proteom. Bioinf..

[17] DAVID Bioinformatics Resources 6.7. https://david-d.ncifcrf.gov/home.jsp.

[18] STRING: Functional Protein Association Networks. http://string-db.org/.

[19] Cancemi P., Di Cara G., Albanese N.N., Costantini F., Marabeti M.R., Musso R., Lupo C., Roz E., Pucci-Minafra I. (2010). Large-scale proteomic identification of S100 proteins in breast cancer tissues. BMC Cancer.

[20] Pucci-Minafra I., Cancemi P., Marabeti M.R., Albanese N.N., Di Cara G., Taormina P., Marrazzo A. (2007). Proteomic profiling of 13 paired ductal infiltrating breast carcinomas and non-tumoral adjacent counterparts. Proteom. Clin. Appl..

[21] Pucci-Minafra I., Cancemi P., Albanese N.N., Di Cara G., Marabeti M.R., Marrazzo A., Minafra S. (2008). New protein clustering of breast cancer tissue proteomics using actin content as a cellularity indicator. J. Proteom. Res..

[22] Pucci-Minafra I., Barh D. (2014). Breast cancer proteomics. Omics Approaches in Breast Cancer.

[23] Warburg O. (1924). Über den stoffwechsel der carcinomzelle. Biochemistry.

[24] Pucci-Minafra I., Fontana S., Cancemi P., Alaimo G., Minafra S. (2002). Proteomic patterns of cultured breast cancer cells and epithelial mammary cells. Ann. N. Y. Acad. Sci..

[25] Li G., Zhao F., Cui Y. (2013). Proteomics using mammospheres as a model system to identify proteins deregulated in breast cancer stem cells. Curr. Mol. Med..

[26] Altenberg B., Greulich K.O. (2004). Genes of glycolysis are ubiquitously overexpressed in 24 cancer classes. Genomics.

[27] Chaerkady R., Harsha H.C., Nalli A., Gucek M., Vivekanandan P., Akhtar J., Cole R.N., Simmers J., Schulick R.D., Singh S. (2008). A quantitative proteomic approach for identification of potential biomarkers in hepatocellular carcinoma. J. Proteom. Res..

[28] Du S., Guan Z., Hao L., Song Y., Wang L., Gong L., Liu L., Qi X., Hou Z., Shao S. (2014). Fructose-bisphosphate aldolase a is a potential metastasis-associated marker of lung squamous cell carcinoma and promotes lung cell tumorigenesis and migration. PLoS ONE.

[29] Poschmann G., Sitek B., Sipos B., Ulrich A., Wiese S., Stephan C., Warscheid B., Klöppel G., Borght A.V., Ramaekers F.C.S. (2009). Identification of proteomic differences between squamous cell carcinoma of the lung and bronchial epithelium. Mol. Cell. Proteom..

[30] Chen X., Yang T.T., Zhou Y., Wang W., Qiu X.C., Gao J., Li C.X., Long H., Ma B.A., Ma Q. (2014). Proteomic profiling of osteosarcoma cells identifies ALDOA and SULT1A3 as negative survival markers of human osteosarcoma. Mol. Carcinog..

[31] Peng Y., Li X., Wu M., Yang J., Liu M., Zhang W., Xiang B., Wang X., Li X., Li G. (2012). New prognosis biomarkers identified by dynamic proteomic analysis of colorectal cancer. Mol. Biosyst..

[32] Kim J.H., Lee S., Kim J.H., Lee T.G., Hirata M., Suh P.G., Ryu S.H. (2002). Phospholipase D2 directly interacts with aldolase via Its PH domain. Biochemistry.

[33] Kao A.W., Noda Y., Johnson J.H., Pessin J.E., Saltiel A.R. (1999). Aldolase mediates the association of F-actin with the insulin-responsive glucose transporter GLUT4. J. Biol. Chem..

[34] Buscaglia C.A., Penesetti D., Tao M., Nussenzweig V. (2006). Characterization of an aldolase-binding site in the Wiskott-Aldrich syndrome protein. J. Biol. Chem..

[35] Lincet H., Icard P. (2015). How do glycolytic enzymes favour cancer cell proliferationby nonmetabolic functions?. Oncogene.

[36] Mamczur P., Gamian A., Kolodziej J., Dziegiel P., Rakus D. (2013). Nuclear localization of aldolase A correlates with cell proliferation. Biochim. Biophys. Acta.

[37] Zhang F., Lin J.D., Zuo X.Y., Zhuang Y.X., Hong C.Q., Zhang G.J., Cui X.J., Cui Y.K. (2017). Elevated transcriptional levels of aldolase A (ALDOA) associates with cell cycle-related genes in patients with NSCLC and several solid tumors. BioData Min..

[38] Guo C., Liu S., Sun M.Z. (2013). Novel insight into the role of GAPDH playing in tumor. Clin. Transl. Oncol..

[39] Demarse N.A., Ponnusamy S., Spicer E.K., Apohan E., Baatz J.E., Ogretmen B., Davies C. (2009). Direct binding of glyceraldehyde 3-phosphate dehydrogenase to telomeric DNA protects telomeres against chemotherapy-induced rapid degradation. J. Mol. Biol..

[40] Carujo S., Estanyol J.M., Ejarque A., Agell N., Bachs O., Pujol M.J. (2006). Glyceraldehyde 3-phosphate dehydrogenase is a SET-binding protein and regulates cyclin B-cdk1 activity. Oncogene.

[41] Inoki K., Li Y., Xu T., Guan K.L. (2003). Rheb GTPase is a direct target of TSC2 GAP activity and regulates mTOR signaling. Genes Dev..

[42] Tristan C., Shahani N., Sedlak T.W., Sawa A. (2011). The diverse functions of GAPDH: Views from different subcellular compartments. Cell. Signal..

[43] Sirover M.A. (2012). Subcellular dynamics of multifunctional protein regulation: Mechanisms of GAPDH intracellular translocation. J. Cell. Biochem..

[44] Huang W., Wang Z., Lei Q.Y. (2014). Acetylation control of metabolic enzymes in cancer: An updated version. Acta Biochim. Biophys. Sin..

[45] Sirover M.A. (2011). On the functional diversity of glyceraldehyde-3-phosphate dehydrogenase: Biochemical mechanisms and regulatory control. Biochim. Biophys. Acta.

[46] Krasnov G.S., Dmitriev A.A., Snezhkina A.V., Kudryavtseva A.V. (2013). Deregulation of glycolysis in cancer: Glyceraldehyde-3-phosphate dehydrogenase as a therapeutic target. Expert Opin. Ther. Targets.

[47] Lee J., Kim H.K., Han Y.M., Kim J. (2008). Pyruvate kinase isozyme type M2 (PKM2) interacts and cooperates with Oct-4 in regulating transcription. Int. J. Biochem. Cell Biol..

[48] Allison S.J., Knight J.R.P., Granchi C., Rani R., Minutolo F., Milner J., Phillips R.M. (2014). Identification of LDH-A as a therapeutic target for cancer cell killing via (I) p53/NAD(H)-dependent and (II) p53-independent pathways. Oncogenesis.

[49] Jin L., Chun J., Pan C., Alesi G.N., Li D., Magliocca K.R., Kang Y., Chen Z.G., Shin D.M., Khuri F.R. (2017). Phosphorylation-mediated activation of LDHA promotes cancer cell invasion and tumour metastasis. Oncogene.

[50] Chang C.C., Zhang C., Zhang Q., Sahin O., Wang H., Xu J., Xiao Y., Zhang J., Rehman S.K., Li P. (2016). Upregulation of lactate dehydrogenase a by 14–3-3ζ leads to increased glycolysis critical for breast cancer initiation and progression. Oncotarget.

[51] Sun S., Liang X., Zhang X., Liu T., Shi Q., Song Y., Jiang Y., Wu H., Jiang Y., Lu X. (2015). Phosphoglycerate kinase-1 is a predictor of poor survival and a novel prognostic biomarker of chemoresistance to paclitaxel treatment in breast cancer. Br. J. Cancer.

[52] Ahmad S.S., Glatzle J., Bajaeifer K., Bühler S., Lehmann T., Königsrainer I., Vollmer J.P., Sipos B., Ahmad S.S., Northoff H. (2013). Phosphoglycerate kinase 1 as a promoter of metastasis in colon cancer. Int. J. Oncol..

[53] Hu H., Zhu W., Qin J., Chen M., Gong L., Li L., Liu X., Tao Y., Yin H., Zhou H. (2017). Acetylation of PGK1 promotes liver cancer cell proliferation and tumorigenesis. Hepatology.

[54] Wang J., Wang J., Dai J., Jung Y., Wei C.L., Wang Y., Havens A.M., Hogg P.J., Keller E.T., Pienta K.J. (2007). A glycolytic mechanism regulating an angiogenic switch in prostate cancer. Cancer Res..

[55] Daly E.B., Wind T., Jiang X.M., Sun L., Hogg P.J. (2004). Secretion of phosphoglycerate kinase from tumour cells is controlled by oxygen-sensing hydroxylases. Biochim. Biophys. Acta.

[56] Vizin T., Kos J. (2015). Gamma-enolase: A well-known tumour marker, with a less-known role in cancer. Radiol. Oncol..

[57] Portt L., Norman G., Clapp C., Greenwood M., Greenwood M.T. (2011). Anti-apoptosis and cell survival: A review. Biochim. Biophys. Acta.

[58] Ichim G., Tait S.W. (2016). A fate worse than death: Apoptosis as an oncogenic process. Nat. Rev. Cancer.

[59] Gene Ontology Consortium. http://www.geneontology.org/.

[60] Wilker E.W., Grant R.A., Artim S.C., Yaffe M.B. (2005). A structural basis for 14-3-3sigma functional specificity. J. Biol. Chem..

[61] Aghazadeh Y., Papadopoulos V. (2016). The role of the 14-3-3 protein family in health, disease, and drug development. Drug Discov. Today.

[62] Ling C., Zuo D., Xue B., Muthuswamy S., Muller W.J. (2010). A novel role for 14-3-3 sigma in regulating epithelial cell polarity. Genes Dev..

[63] Freeman A.K., Morrison D.K. (2011). 14-3-3 Proteins: Diverse functions in cell proliferation and cancer progression. Semin. Cell Dev. Biol..

[64] Masters S.C., Fu H. (2001). 14-3-3 proteins mediate an essential anti-apoptotic signal. J. Biol. Chem..

[65] Rosenquist M. (2003). 14-3-3 proteins in apoptosis. Braz. J. Med. Biol. Res..

[66] Lee J.H., Lu H. (2011). 14-3-3 Gamma inhibition of MDMX-mediated p21 turnover independent of p53. J. Biol. Chem..

[67] Gerke V., Creutz C.E., Moss S.E. (2005). Annexins: Linking Ca^2+^ signalling to membrane dynamics. Nat. Rev. Mol. Cell Biol..

[68] Mussunoor S., Murray G.I. (2008). The role of annexins in tumour development and progression. J. Pathol..

[69] Wehder L., Arndt S., Murzik U., Bosserhoff A.K., Kob R., von Eggeling F., Melle C. (2009). Annexin A5 is involved in migration and invasion of oral carcinoma. Cell Cycle.

[70] Wang R.C., Huang C.Y., Pan T.L., Chen W.Y., Ho C.T., Liu T.Z., Chang Y.J. (2015). Proteomic characterization of annexin l (ANX1) and heat shock protein 27 (HSP27) as biomarkers for invasive hepatocellular carcinoma cells. PLoS ONE.

[71] Peng B., Guo C., Guan H., Liu S., Sun M.Z. (2014). Annexin A5 as a potential marker in tumors. Clin. Chim. Acta.

[72] Rezvani H.R., Mazurier F., Cario-André M., Pain C., Ged C., Taïeb A., de Verneuil H. (2006). Protective effects of catalase overexpression on UVB-induced apoptosis in normal human keratinocytes. J. Biol. Chem..

[73] Kanellos G., Frame M.C. (2016). Cellular functions of the ADF/cofilin family at a glance. J. Cell Sci..

[74] Wang C., Zhou G., Vedantam S., Li P., Field J. (2008). Mitochondrial shuttling of CAP1 promotes actin- and cofilin-dependent apoptosis. J. Cell Sci..

[75] Bruneel A., Labas V., Mailloux A., Sharma S., Royer N., Vinh J., Pernet P., Vaubourdolle M., Baudin B. (2005). Proteomics of human umbilical vein endothelial cells applied to etoposide-induced apoptosis. Proteomics.

[76] Rehklau K., Gurniak C.B., Conrad M., Friauf E., Ott M., Rust M.B. (2012). ADF/cofilin proteins translocate to mitochondria during apoptosis but are not generally required for cell death signaling. Cell Death Differ..

[77] Maimaiti Y., Liu Z., Tan J., Abudureyimu K., Huang B., Liu C., Guo Y., Wang C., Nie X., Zhou J., Huang T. (2016). Dephosphorylated cofilin expression is associated with poor prognosis in cases of human breast cancer: A tissue microarray analysis. OncoTargets Ther..

[78] Jolly C., Morimoto R.I. (2000). Role of the heat shock response and molecular chaperones in oncogenesis and cell death. J. Natl. Cancer Inst..

[79] Bakthisaran R., Tangirala R., Rao C.M. (2015). Small heat shock proteins: Role in cellular functions and pathology. Biochim. Biophys. Acta.

[80] Wu J., Liu T., Rios Z., Mei Q., Lin X., Cao S. (2017). Heat shock proteins and cancer. Trends Pharmacol. Sci..

[81] Ciocca D.R., Calderwood S.K. (2005). Heat shock proteins in cancer: Diagnostic, prognostic, predictive, and treatment implications. Cell Stress Chaperones.

[82] Ağababaoğlu İ., Önen A., Demir A.B., Aktaş S., Altun Z., Ersöz H., Şanl A., Özdemir N., Akkoçlu A. (2017). Chaperonin (HSP60) and annexin-2 are candidate biomarkers for non-small cell lung carcinoma. Medicine.

[83] Zhou H., Zhang Y., Fu Y., Chan L., Lee A.S. (2011). Novel mechanism of anti-apoptotic function of 78-kDa glucose-regulated protein (GRP78): Endocrine resistance factor in breast cancer, through release of B-cell lymphoma 2 (BCL-2) from BCL-2-interacting killer (BIK). J. Biol. Chem..

[84] Spike B.T., Kelber J.A., Booker E., Kalathur M., Rodewald R., Lipianskaya J., La J., He M., Wright T., Klemke R. (2014). CRIPTO/GRP78 signaling maintains fetal and adult mammary stem cells Ex Vivo. Stem Cell Rep..

[85] Gray P.C., Vale W. (2012). Cripto/GRP78 modulation of the TGF-β pathway in development and oncogenesis. FEBS Lett..

[86] Wu S., Hong F., Gewirth D., Guo B., Liu B., Li Z. (2012). The molecular chaperone gp96/GRP94 interacts with Toll-like Receptors and Integrins via its C-terminal hydrophobic domain. J. Biol. Chem..

[87] Wu B.X., Hong F., Zhang Y., Ansa-Addo E., Li Z. (2016). GRP94/gp96 in cancer: Biology, structure, immunology, and drug development. Adv. Cancer Res..

[88] Kawatani M., Okumura H., Honda K., Kanoh N., Muroi M., Dohmae N., Takami M., Kitagawa M., Futamura Y., Imoto M. (2008). The identification of an osteoclastogenesis inhibitor through the inhibition of glyoxalase I. Proc. Natl. Acad. Sci. USA.

[89] Conroy H., Mawhinney L., Donnelly S.C. (2010). Inflammation and cancer: Macrophage migration inhibitory factor (MIF)—The potential missing link. Q. J. Med..

[90] Nobre C., de Araújo J.M., Fernandes T.A., Cobucci R.N., Lanza D.C., Andrade V.S., Fernandes J.V. (2016). Macrophage migration inhibitory factor (MIF): Biological activities and relation with cancer. Pathol. Oncol. Res..

[91] Richard V., Kindt N., Saussez S. (2015). Macrophage migration inhibitory factor involvement in breast cancer (Review). Int. J. Oncol..

[92] Steeg P.S. (1998). Breast cancer advocacy and basic research: A scientist’s perspective. Breast Dis..

[93] Bevilacqua G., Sobel M.E., Liotta L.A., Steeg P.S. (1989). Association of low nm23 RNA levels in human primary infiltrating ductal breast carcinomas with lymph node involvement and other histopathological indicators of high metastatic potential. Cancer Res..

[94] Yan J., Yang Q., Huang Q. (2013). Metastasis suppressor genes. Histol. Histopathol..

[95] Box J.K., Paquet N., Adams M.N., Boucher D., Bolderson E., O’Byrne K.J., Richard D.J. (2016). Nucleophosmin: From structure and function to disease development. BMC Mol. Biol..

[96] Pang Q., Christianson T.A., Koretsky T., Carlson H., David L., Keeble W., Faulkner G.R., Speckhart A., Bagby G.C. (2003). Nucleophosmin interacts with and inhibits the catalytic function of eukaryotic initiation factor 2 kinase PKR. J. Biol. Chem..

[97] Gasser T., Müller-Myhsok B., Wszolek Z.K., Dürr A., Vaughan J.R., Bonifati V., Meco G., Bereznai B., Oehlmann R., Agid Y. (1997). Genetic complexity and Parkinson’s disease. Science.

[98] Vasseur S., Afzal S., Tardivel-Lacombe J., Park D.S., Iovanna J.L., Mak T.W. (2009). DJ-1/PARK7 is an important mediator of hypoxia-induced cellular responses. Proc. Natl. Acad. Sci. USA.

[99] Kim R.H., Peters M., Jang Y., Shi W., Pintilie M., Fletcher G.C., De Luca C., Liepa J., Zhou L., Snow B. (2005). DJ-1, a novel regulator of the tumor suppressor PTEN. Cancer Cell.

[100] Davidson B., Hadar R., Schlossberg A., Sternlicht T., Slipicevic A., Skrede M., Risberg B., Flørenes V.A., Kopolovic J., Reich R. (2008). Expression and clinical role of DJ-1, a negative regulator of PTEN, in ovarian carcinoma. Hum. Pathol..

[101] Zhang S., Mukherjee S., Fan X., Salameh A., Mujoo K., Huang Z., Li L., To’a Salazar G., Zhang N., An Z. (2016). Novel association of DJ-1 with HER3 potentiates HER3 activation and signaling in cancer. Oncotarget.

[102] Dasgupta J., Subbaram S., Connor K.M., Rodriguez A.M., Tirosh O., Beckman J.S., Jourd’Heuil D., Melendez J.A. (2006). Manganese superoxide dismutase protects from TNF-alpha-induced apoptosis by increasing the steady-state production of H_2_O_2_. Antioxid. Redox Signal..

[103] Bae S.Y., Kim H.J., Lee K.J., Lee K. (2015). Translationally controlled tumor protein induces epithelial to mesenchymal transition and promotes cell migration, invasion and metastasis. Sci. Rep..

[104] Chan T.H., Chen L., Guan X.Y. (2012). Role of translationally controlled tumor protein in cancer progression. Biochem. Res. Int..

[105] Karlenius T.C., Tonissen K.F. (2010). Thioredoxin and cancer: A Role for thioredoxin in all states of tumor oxygenation. Cancers.

[106] Fan J., Yu H., Lu Y., Yin L. (2016). Diagnostic and prognostic value of serum thioredoxin and DJ-1 in non-small cell lung carcinoma patients. Tumour Biol..

[107] Raninga P.V., Trapani G.D., Tonissen K.F. (2014). Cross talk between two antioxidant systems, thioredoxin and DJ-1: Consequences for cancer. Oncoscience.

[108] Cheng E.H., Sheiko T.V., Fisher J.K., Craigen W.J., Korsmeyer S.J. (2003). VDAC2 inhibits BAK activation and mitochondrial apoptosis. Science.

[109] HGNC (HUGO Gene Nomenclature Committee). http://www.genenames.org/cgi-bin/genefamilies/set/459.

[110] Salama I., Malone P.S., Mihaimeed F., Jones J.L. (2008). A review of the S100 proteins in cancer. Eur. J. Surg. Oncol..

[111] Donato R., Cannon B.R., Sorci G., Riuzzi F., Hsu K., Weber D.J., Geczy C.L. (2013). Functions of S100 proteins. Curr. Mol. Med..

[112] Leclerc E., Fritz G., Vetter S.W., Heizmann C.W. (2009). Binding of S100 proteins to RAGE: An update. Biochim. Biophys. Acta.

[113] Hsu K., Passey R.J., Endoh Y., Rahimi F., Youssef P., Yen T., Geczy C.L. (2005). Regulation of S100A8 by glucocorticoids. J. Immunol..

[114] Gläser R., Harder J., Lange H., Bartels J., Christophers E., Schröder J.M. (2005). Antimicrobial psoriasin (S100A7) protects human skin from Escherichia coli infection. Nat. Immunol..

[115] Bresnick A.R., Weber D.J., Zimmer D.B. (2015). S100 proteins in cancer. Nat. Rev. Cancer.

[116] Tsai W.C., Tsai S.T., Jin Y.T., Wu L.W. (2006). Cyclooxygenase-2 is involved in S100A2-mediated tumor suppression in squamous cell carcinoma. Mol. Cancer Res..

[117] Bulk E., Sargin B., Krug U., Hascher A., Jun Y., Knop M., Kerkhoff C., Gerke V., Liersch R., Mesters R.M. (2009). S100A2 induces metastasis in non-small cell lung cancer. Clin. Cancer Res..

[118] Yamaguchi F., Umeda Y., Shimamoto S., Tsuchiya M., Tokumitsu H., Tokuda M., Kobayashi R. (2012). S100 proteins modulate protein phosphatase 5 function: A link between CA^2+^ signal transduction and protein dephosphorylation. J. Biol. Chem..

[119] Anania M.C., Miranda C., Vizioli M.G., Mazzoni M., Cleris L., Pagliardini S., Manenti G., Borrello M.G., Pierotti M.A., Greco A. (2013). S100A11 overexpression contributes to the malignant phenotype of papillary thyroid carcinoma. J. Clin. Endocrinol. Metab..

[120] Hao J., Wang K., Yue Y., Tian T., Xu A., Hao J., Xiao X., He D. (2012). Selective expression of S100A11 in lung cancer and its role in regulating proliferation of adenocarcinomas cells. Mol. Cell. Biochem..

[121] Pierce A., Barron N., Linehan R., Ryan E., O’Driscoll L., Daly C., Clynes M. (2008). Identification of a novel, functional role for S100A13 in invasive lung cancer cell lines. Eur. J. Cancer.

[122] Massi D., Landriscina M., Piscazzi A., Cosci E., Kirov A., Paglierani M., Di Serio C., Mourmouras C., Santucci M., Marchionni N. (2010). S100A13 is a new angiogenic marker in human melanoma. Mod. Pathol..

[123] Sapkota D., Bruland O., Parajuli H., Osman T.A., Teh M., Johannessen A.C., Costea D.E. (2015). S100A16 promotes differentiation and contributes to a less aggressive tumor phenotype in oral squamous cell carcinoma. BMC Cancer.

[124] Zhu W., Xue Y., Liang C., Zhang R., Zhang Z., Li H., Su D., Liang X., Zhang Y., Huang Q. (2016). S100A16 promotes cell proliferation and metastasis via AKT and ERK cell signaling pathways in human prostate cancer. Tumour. Biol..

[125] Tanaka M., Ichikawa-Tomikawa N., Shishito N., Nishiura K., Miura T., Hozumi A., Chiba H., Yoshida S., Ohtake T., Sugino T. (2015). Co-expression of S100A14 and S100A16 correlates with a poor prognosis in human breast cancer and promotes cancer cell invasion. BMC Cancer.

[126] Chen L., Brewer M.D., Guo L., Wang R., Jiang P., Yang X. (2017). Enhanced degradation of misfolded proteins promotes tumorigenesis. Cell Rep..

[127] Barrière G., Tartary M., Rigaud M. (2012). Epithelial mesenchymal transition: A new insight into the detection of circulating tumor cells. ISRN Oncol..

[128] Pucci-Minafra I., Minafra S., La Rocca G., Barranca M., Fontana S., Alaimo G., Okada Y. (2001). Zymographic analysis of circulating and tissue forms of colon carcinoma gelatinase A (MMP-2) and B (MMP-9) separated by mono- and two-dimensional electrophoresis. Matrix Biol..

[129] Pollard T.D., Borisy G.G. (2003). Cellular motility driven by assembly and disassembly of actin filaments. Cell.

[130] Smith B.A., Daugherty-Clarke K., Goode B.L., Gelles J. (2013). Pathway of actin filament branch formation by Arp2/3 complex revealed by single-molecule imaging. Proc. Natl. Acad. Sci. USA.

[131] Yamazaki D., Kurisu S., Takenawa T. (2005). Regulation of cancer cell motility through actin reorganization. Cancer Sci..

[132] Kinoshita T., Nohata N., Watanabe-Takano H., Yoshino H., Hidaka H., Fujimura L., Fuse M., Yamasaki T., Enokida H., Nakagawa M. (2012). Actin-related protein 2/3 complex subunit 5 (ARPC5) contributes to cell migration and invasion and is directly regulated by tumor-suppressive microRNA-133a in head and neck squamous cell carcinoma. Int. J. Oncol..

[133] Zhang H., Zhou G.L. (2016). CAP1 (cyclase-associated protein 1) exerts distinct functions in the proliferation and metastatic potential of breast cancer cells mediated by ERK. Sci. Rep..

[134] Westbrook J.A., Cairns D.A., Peng J., Speirs V., Hanby A.M., Holen I., Wood S.L., Ottewell P.D., Marshall H., Banks R.E. (2016). CAPG and GIPC1: Breast cancer biomarkers for bone metastasis development and treatment. J. Natl. Cancer Inst..

[135] Chan C., Beltzner C.C., Pollard T.D. (2009). Cofilin dissociates Arp2/3 complex and branches from actin filaments. Curr. Biol..

[136] Zhou G.L., Zhang H., Field J. (2014). Mammalian CAP (Cyclase-associated protein) in the world of cell migration: Roles in actin filament dynamics and beyond. Cell Adh. Migr..

[137] Bravo-Cordero J.J., Magalhaes M.A., Eddy R.J., Hodgson L., Condeelis J. (2013). Functions of cofilin in cell locomotion and invasion. Nat. Rev. Mol. Cell Biol..

[138] Hou X., Katahira T., Ohashi K., Mizuno K., Sugiyama S., Nakamura H. (2013). Coactosin accelerates cell dynamism by promoting actin polymerization. Dev. Biol..

[139] Sun W., Guo C., Meng X., Yu Y., Jin Y., Tong D., Geng J., Huang Q., Qi J., Liu A. (2012). Differential expression of PAI-RBP1, C1orf142, and COTL1 in non-small cell lung cancer cell lines with different tumor metastatic potential. J. Investig. Med..

[140] Hoskin V., Szeto A., Ghaffari A., Greer P.A., Côté G.P., Elliott B.E. (2015). Ezrin regulates focal adhesion and invadopodia dynamics by altering calpain activity to promote breast cancer cell invasion. Mol. Biol. Cell.

[141] Asp N., Kvalvaag A., Sandvig K., Pust S. (2016). Regulation of ErbB2 localization and function in breast cancer cells by ERM proteins. Oncotarget.

[142] Shetty P., Bargale A., Patil B.R., Mohan R., Dinesh U.S., Vishwanatha J.K., Gai P.B., Patil V.S., Amsavardani T.S. (2016). Cell surface interaction of annexin A2 and galectin-3 modulates epidermal growth factor receptor signaling in Her-2 negative breast cancer cells. Mol. Cell. Biochem..

[143] Fukumori T., Takenaka Y., Yoshii T., Kim H.R., Hogan V., Inohara H., Kagawa S., Raz A. (2003). CD29 and CD7 mediate galectin-3-induced type II T-cell apoptosis. Cancer Res..

[144] De Oliveira F.L., Gatto M., Bassi N., Luisetto R., Ghirardello A., Punzi L., Doria A. (2015). Galectin-3 in autoimmunity and autoimmune diseases. Exp. Biol. Med..

[145] Zhang H., Luo M., Liang X., Wang D., Gu X., Duan C., Gu H., Chen G., Zhao X., Zhao Z. (2014). Galectin-3 as a marker and potential therapeutic target in breast cancer. PLoS ONE.

[146] Gohla A., Birkenfeld J., Bokoch G.M. (2005). Chronophin, a novel HAD-type serine protein phosphatase, regulates cofilin-dependent actin dynamics. Nat. Cell Biol..

[147] Cao X.X., Xu J.D., Xu J.W., Liu X.L., Cheng Y.Y., Li Q.Q., Xu Z.D., Liu X.P. (2011). RACK1 promotes breast carcinoma migration/metastasis via activation of the RhoA/Rho kinase pathway. Breast Cancer Res. Treat..

[148] Emery G., Ramel D. (2013). Cell coordination of collective migration by Rab11 and Moesin. Commun. Integr. Biol..

[149] Kuang X.Y., Jiang H.S., Li K., Zheng Y.Z., Liu Y.R., Qiao F., Li S., Hu X., Shao Z.M. (2016). The phosphorylation-specific association of STMN1 with GRP78 promotes breast cancer metastasis. Cancer Lett..

[150] Fessart D., Domblides C., Avril T., Eriksson L.A., Begueret H., Pineau R., Malrieux C., Dugot-Senant N., Lucchesi C., Chevet E. (2016). Secretion of protein disulphide isomerase AGR2 confers tumorigenic properties. eLife.

[151] Ondrouskova E., Sommerova L., Nenutil R., Coufal O., Bouchal P., Vojtesek B., Hrstka R. (2017). AGR2 associates with HER2 expression predicting poor outcome in subset of estrogen receptor negative breast cancer patients. Exp. Mol. Pathol..

[152] Dumartin L., Whiteman H.J., Weeks M.E., Hariharan D., Dmitrovic B., Iacobuzio-Donahue C.A., Brentnall T.A., Bronner M.P., Feakins R.M., Timms J.F. (2011). AGR2 is a novel surface antigen that promotes the dissemination of pancreatic cancer cells through regulation of cathepsins B and D. Cancer Res..

[153] Alavi M., Mah V., Maresh E.L., Bagryanova L., Horvath S., Chia D., Goodglick L., Liu A.Y. (2015). High expression of AGR2 in lung cancer is predictive of poor survival. BMC Cancer.

[154] Arya R., Mallik M., Lakhotia S.C. (2007). Heat shock genes-integrating cell survival and death. J. Biosci..

[155] Wadhwa R., Takano S., Kaur K., Deocaris C.C., Pereira-Smith O.M., Reddel R.R., Kaul S.C. (2006). Upregulation of mortalin/mthsp70/Grp75 contributes to human carcinogenesis. Int. J. Cancer.

[156] Jin H., Ji M., Chen L., Liu Q., Che S., Xu M., Lin Z. (2016). The clinicopathological significance of Mortalin overexpression in invasive ductal carcinoma of breast. J. Exp. Clin. Cancer Res..

[157] Zhou Y., Liao Q., Li X., Wang H., Wei F., Chen J., Yang J., Zeng Z., Guo X., Chen P. (2016). HYOU1, regulated by LPLUNC1, is up-regulated in nasopharyngeal carcinoma and associated with poor prognosis. J. Cancer.

[158] Jarosz D. (2016). Hsp90: A global regulator of the genotype-to-phenotype map in cancers. Adv. Cancer Res..

[159] Calderwood S.K., Neckers L. (2016). Hsp90 in Cancer: Transcriptional roles in the nucleus. Adv. Cancer Res..

[160] Fang F., Flegler A.J., Du P., Lin S., Clevenger C.V. (2009). Expression of cyclophilin B is associated with malignant progression and regulation of genes implicated in the pathogenesis of breast cancer. Am. J. Pathol..

[161] Fang F., Zheng J., Galbaugh T.L., Fiorillo A.A., Hjort E.E., Zeng X., Clevenger C.V. (2010). Cyclophilin B as a co-regulator of prolactin-induced gene expression and function in breast cancer cells. J. Mol. Endocrinol..

[162] Javadov S., Kuznetsov A. (2013). Mitochondrial permeability transition and cell death: The role of cyclophilin d. Front. Physiol..

[163] Bigi A., Beltrami E., Trinei M., Stendardo M., Pelicci P.G., Giorgio M. (2016). Cyclophilin D counteracts P53-mediated growth arrest and promotes Ras tumorigenesis. Oncogene.

[164] Millán-Zambrano G., Chávez S. (2014). Nuclear functions of prefoldin. Open Biol..

[165] López V., González-Peramato P., Suela J., Serrano A., Algaba F., Cigudosa J.C., Vidal A., Bellmunt J., Heredero O., Sánchez-Carbayo M. (2013). Identification of prefoldin amplification (1q23.3-q24.1) in bladder cancer using comparative genomic hybridization (CGH) arrays of urinary DNA. J. Transl. Med..

[166] Vreeland A.C., Levi L., Zhang W., Berry D.C., Noy N. (2014). Cellular retinoic acid-binding protein 2 inhibits tumor growth by two distinct mechanisms. J. Biol. Chem..

[167] Yu S., Parameswaran N., Li M., Wang Y., Jackson M.W., Liu H., Xin W., Zhou L. (2016). CRABP-II enhances pancreatic cancer cell migration and invasion by stabilizing interleukin 8 expression. Oncotarge.

[168] Tsai C.L., Chao A., Jung S.M., Tsai C.N., Lin C.Y., Chen S.H., Sue S.C., Wang T.H., Wang H.S., Lai C.H. (2016). Stress-induced phosphoprotein-1 maintains the stability of JAK2 in cancer cells. Oncotarget.

[169] Guest S.T., Kratche Z.R., Bollig-Fischer A., Haddad R., Ethier S.P. (2015). Two members of the TRiC chaperonin complex, CCT2 and TCP1 are essential for survival of breast cancer cells and are linked to driving oncogenes. Exp. Cell Res..

[170] Boudiaf-Benmammar C., Cresteil T., Melki R. (2013). The cytosolic chaperonin CCT/TRiC and cancer cell proliferation. PLoS ONE.

[171] Fuentes M.K., Nigavekar S.S., Arumugam T., Logsdon C.D., Schmidt A.M., Park J.C., Huang EH. (2007). RAGE activation by S100P in colon cancer stimulates growth, migration, and cell signaling pathways. Dis. Colon Rectum.

[172] Arumugam T., Simeone D.M., Van Golen K., Logsdon C.D. (2005). S100P promotes pancreatic cancer growth, survival, and invasion. Clin. Cancer Res..

[173] Hernández J.L., Padilla L., Dakhel S., Coll T., Hervas R., Adan J., Masa M., Mitjans F., Martinez J.M., Coma S. (2013). Therapeutic targeting of tumor growth and angiogenesis with a novel anti-S100A4 monoclonal antibody. PLoS ONE.

[174] Cancemi P., Di Cara G., Albanese N.N., Costantini F., Marabeti M.R., Musso R., Riili I., Lupo C., Roz E., Pucci-Minafra I. (2012). Differential occurrence of S100A7 in breast cancer tissues: A proteomic-based investigation. Proteom. Clin. Appl..

[175] Mandal S., Curtis L., Pind M., Murphy L.C., Watson P.H. (2007). S100A7 (psoriasin) influences immune response genes in human breast cancer. Exp. Cell Res..

[176] West N.R., Watson P.H. (2010). S100A7 (psoriasin) oncostatin-M and interleukin-6 in human breast cancer. Oncogene.

[177] Nasser M.W., Qamri Z., Deol Y.S., Ravi J., Powell C.A., Trikha P., Schwendener R.A., Bai X.F., Shilo K., Zou X. (2012). S100A7 enhances mammary tumorigenesis through upregulation of inflammatory pathways. Cancer Res..

[178] Chen H., Xu C., Jin Q., Liu Z. (2014). S100 protein family in human cancer. Am. J. Cancer Res..

[179] Oberley T.D. (2002). Oxidative damage and cancer. Am. J. Pathol..

[180] Matkowskyj K.A., Bai H., Liao J., Zhang W., Li H., Rao S., Omary R., Yang G.Y. (2014). Aldoketoreductase family 1B10 (AKR1B10) as a biomarker to distinguish hepatocellular carcinoma from benign liver lesions. Hum. Pathol..

[181] Wu L., Shen Y., Peng X., Zhang S., Wang M., Xu G., Zheng X., Wang J., Lu C. (2016). Aberrant promoter methylation of cancer-related genes in human breast cancer. Oncol. Lett..

[182] Zhang J.Y., Zhang F., Hong C.Q., Giuliano A.E., Cui X.J., Zhou G.J., Zhang G.J., Cui Y.K. (2015). Critical protein GAPDH and its regulatory mechanisms in cancer cells. Cancer Biol. Med..

[183] Chung Y.T., Matkowskyj K.A., Li H., Bai H., Zhang W., Tsao M.S., Liao J., Yang G.Y. (2012). Overexpression and oncogenic function of aldo-keto reductase family 1B10 (AKR1B10) in pancreatic carcinoma. Mod. Pathol..

