# Supplementary material for: Retrospective Proteomic Screening of 100 Breast Cancer Tissues
Source: Proteomes. 2017 Jul 7;5(3):15. doi: 10.3390/proteomes5030015 (PMC5620532; doi:10.3390/proteomes5030015)
Supplement: Supplementary file 1 [file proteomes-05-00015-s001.zip › Supplementary Files/SUPPLEMENTARY LEGENDS .docx]

**SUPPLEMENTARY LEGENDS**

**Table S1.** Catalogue of the 453 proteins identified in the breast cancer tissues. The table reports the following information: Abbreviated Names; Accession Numbers (AC) of the Swiss-Prot database; MALDI-TOF parameters and experimental isoelectric point (p*I*) and molecular weight (MW).

**Figure S1.** Diagrams illustrating the occurrence of the 453 identified proteins (ordinate axis) among the 100 studied patients.(abscissa axis). Proteins are indicated according to the Swiss-Prot/TrEMBL database. Isoforms are reported with alphabetical letters. The white boxes indicate the presence of a given protein (or isoform) and , the grey boxes its absence in the corresponding patient map.
