# Supplementary figures and images for: Retrospective Proteomic Screening of 100 Breast Cancer Tissues
Source: Proteomes. 2017 Jul 7;5(3):15. doi: 10.3390/proteomes5030015 (PMC5620532; doi:10.3390/proteomes5030015)

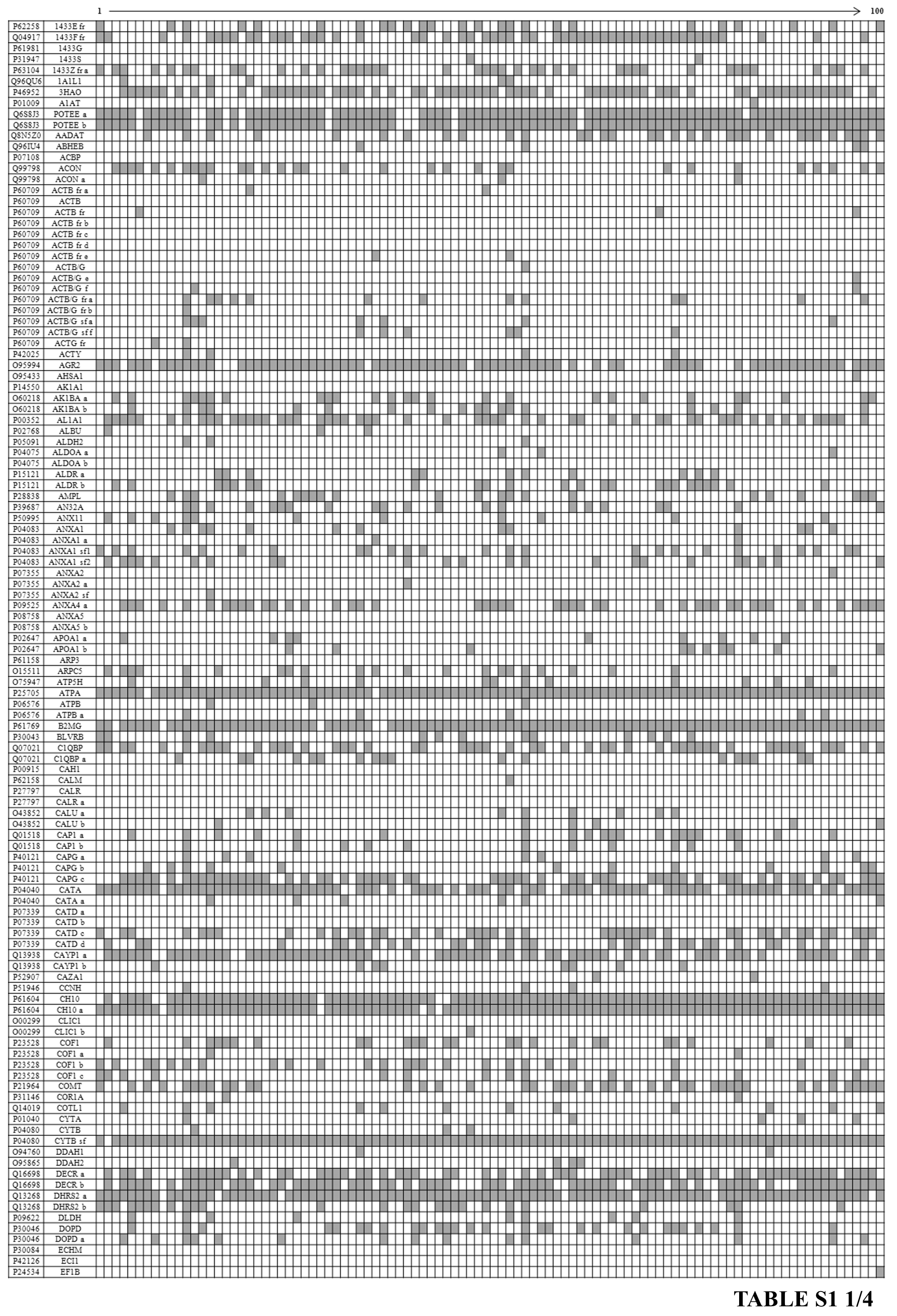

Supplement: Supplementary file 1 [file proteomes-05-00015-s001.zip › Supplementary Files/Figure S1/Figure S1 part 1.TIF]

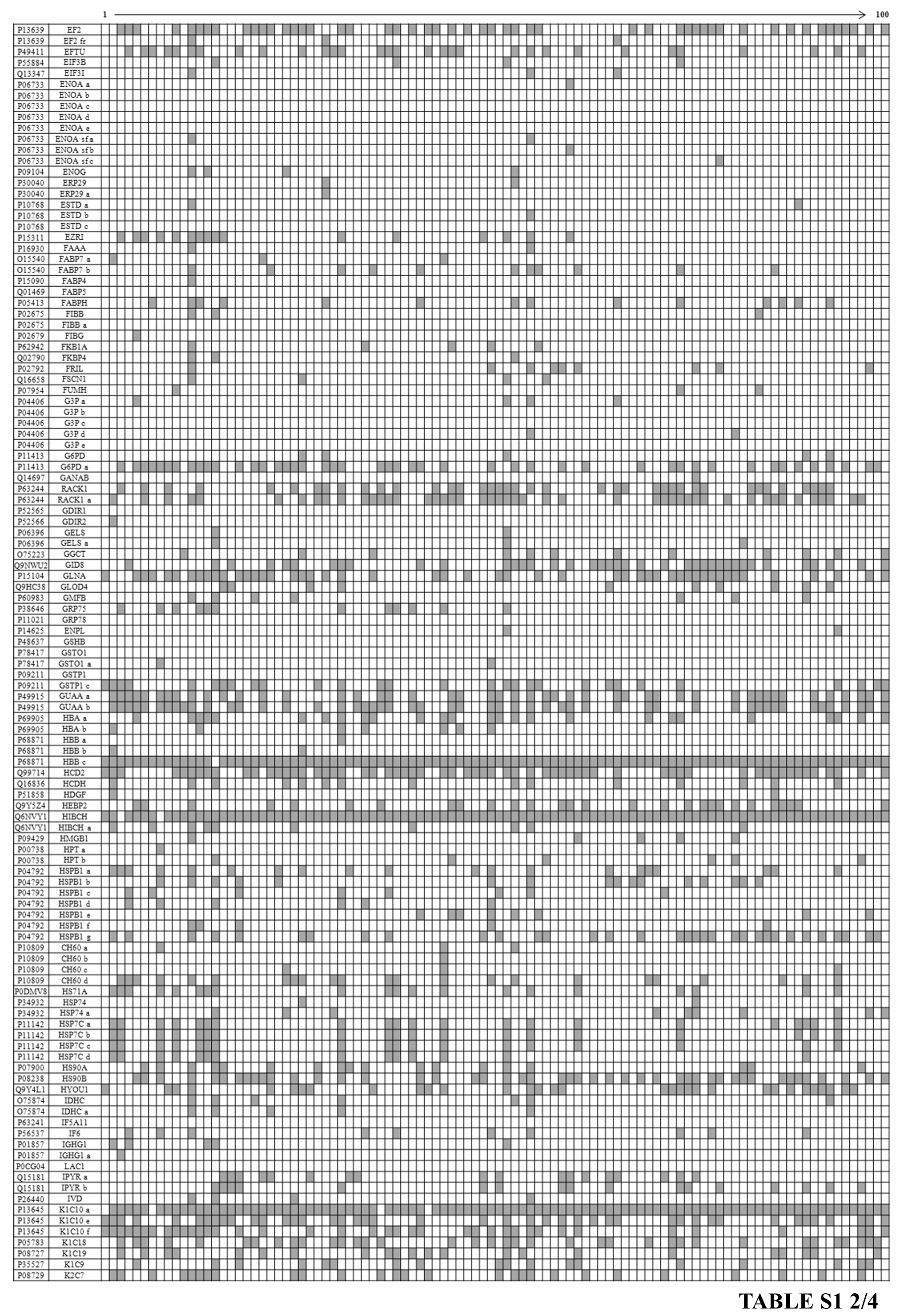

Supplement: Supplementary file 1 [file proteomes-05-00015-s001.zip › Supplementary Files/Figure S1/Figure S1 part 2.TIF]

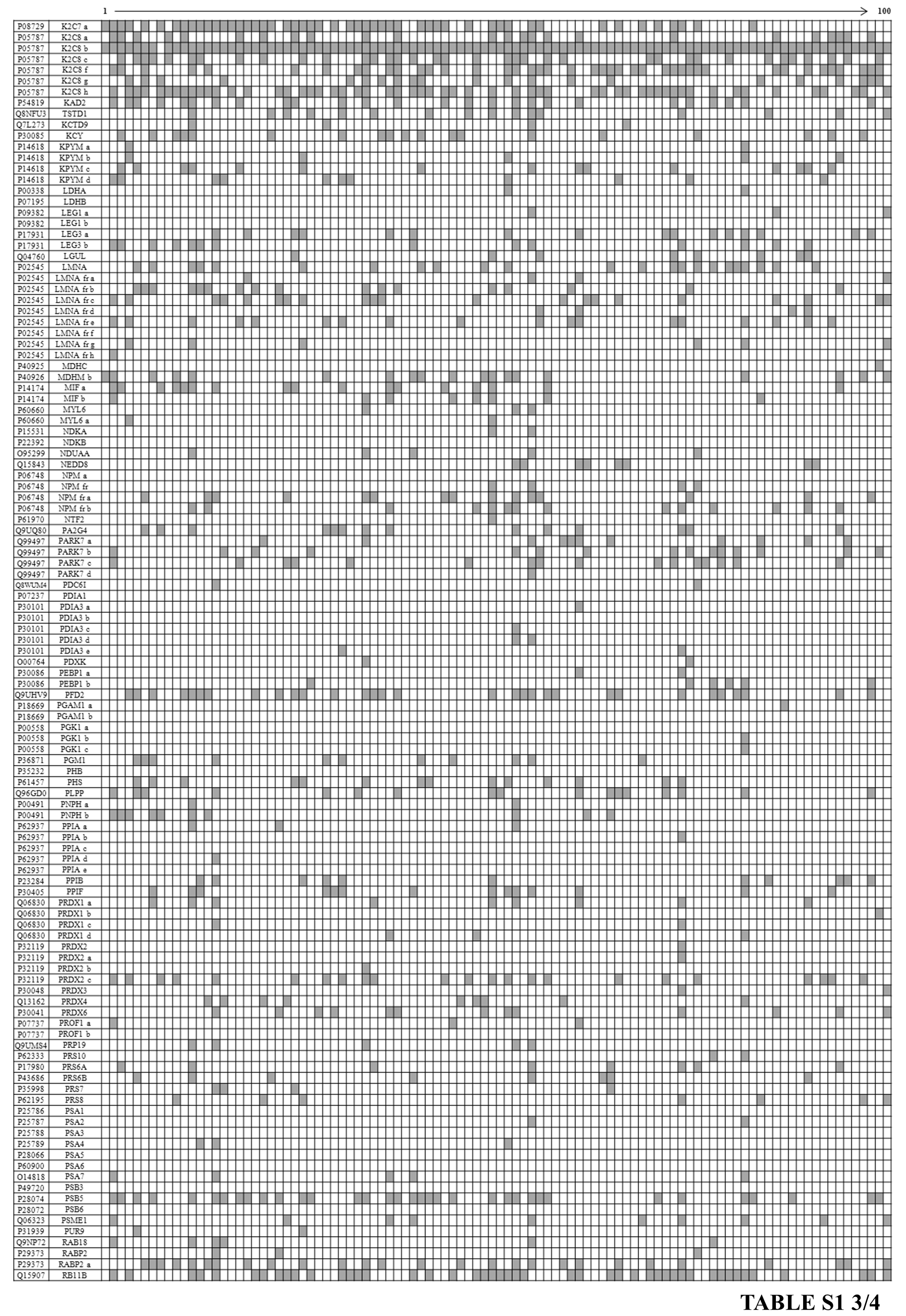

Supplement: Supplementary file 1 [file proteomes-05-00015-s001.zip › Supplementary Files/Figure S1/Figure S1 part 3.TIF]

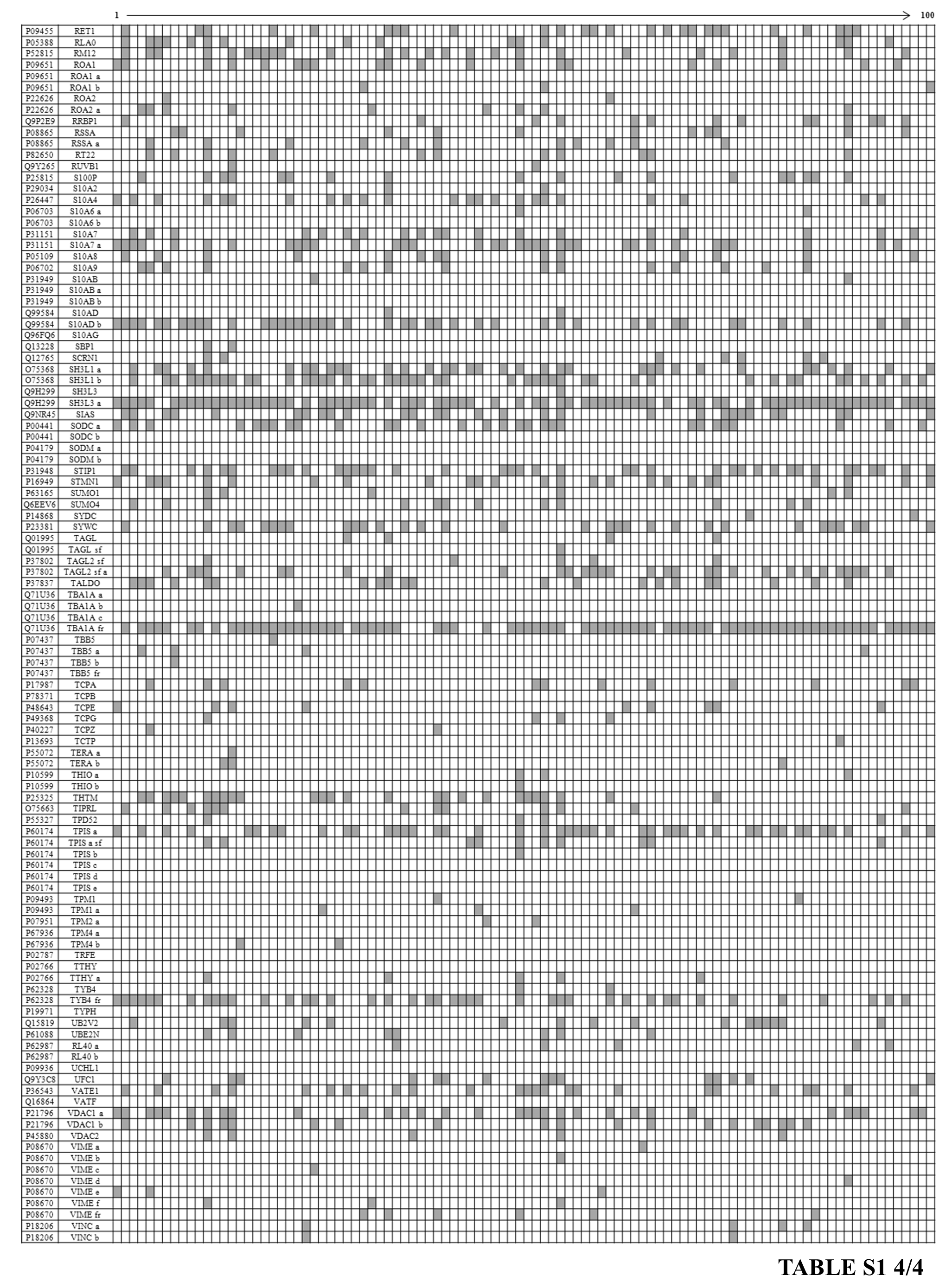

Supplement: Supplementary file 1 [file proteomes-05-00015-s001.zip › Supplementary Files/Figure S1/Figure S1 part 4.TIF]
