# Supplementary material for: Retrospective Proteomic Screening of 100 Breast Cancer Tissues
Source: Proteomes. 2017 Jul 7;5(3):15. doi: 10.3390/proteomes5030015 (PMC5620532; doi:10.3390/proteomes5030015)
Supplement: Supplementary file 1 [file proteomes-05-00015-s001.zip › Supplementary Files/Table S1.doc]

| **Abbreviated**  **TABLE S1**  **Name** | **Uniprot**  **AC** | **Mowse**  **Score** | **Number of mass**  **values matched** | **Number of mass**  **values searched** | **% Masses**  **Matched** | **Seq.**  **Coverage**  **(%)** | **pI**  **exp.** | **MW (Da)**  **exp.** |
| --- | --- | --- | --- | --- | --- | --- | --- | --- |
| 1433E fr | P62258 | 61 | 11 | 48 | 22.92 | 27 | 5.03 | 20517 |
| 1433F fr | Q04917 | 64 | 13 | 69 | 18.84 | 38 | 5.81 | 21214 |
| 1433G | P61981 | 174 | 14 | 18 | 77.78 | 41 | 4.75 | 27437 |
| 1433S | P31947 | 74 | 13 | 71 | 18.31 | 32 | 4.70 | 27266 |
| 1433Z fr | P63104 | 115 | 13 | 55 | 23.64 | 39 | 5.24 | 20823 |
| 3HAO | P46952 | 60 | 11 | 82 | 13.41 | 45 | 5.82 | 82857 |
| A1AT | P01009 | 95 | 12 | 43 | 27.90 | 30 | 5.49 | 25216 |
| 1A1L1 | Q96QU6 | 66 | 6 | 14 | 42.86 | 14 | 4.84 | 54766 |
| AADAT | Q8N5Z0 | 70 | 6 | 11 | 54.55 | 10 | 6.82 | 43753 |
| ABHEB | Q96IU4 | 57 | 4 | 12 | 33.33 | 22 | 5.82 | 23086 |
| ACBP | P07108 | 75 | 10 | 60 | 16.67 | 72 | 5.79 | 10897 |
| ACON | Q99798 | 73 | 8 | 21 | 38.10 | 12 | 6.86 | 95409 |
| ACON a | Q99798 | 73 | 8 | 21 | 38.10 | 12 | 7.27 | 83493 |
| ACTB fr a | P60709 | 64 | 7 | 22 | 31.82 | 20 | 5.47 | 30602 |
| ACTB | P60709 | 68 | 18 | 34 | 13.43 | 57 | 5.09 | 43367 |
| ACTB fr | P60709 | 59 | 5 | 10 | 50.00 | 13 | 5.35 | 32178 |
| ACTB fr b | P60709 | 63 | 7 | 23 | 30.43 | 26 | 5.35 | 26510 |
| ACTB fr c | P60709 | 64 | 5 | 12 | 41.67 | 13 | 5.33 | 27782 |
| ACTB fr d | P60709 | 75 | 6 | 8 | 75.00 | 18 | 5.20 | 27036 |
| ACTB fr e | P60709 | 70 | 6 | 24 | 25.00 | 22 | 4.93 | 25930 |
| ACTB/G | P60709 | 71 | 8 | 34 | 23.53 | 20 | 5.64 | 40638 |
| ACTB/G e | P60709 | 56 | 7 | 54 | 12.96 | 20 | 5.20 | 43367 |
| ACTB/G f | P60709 | 115 | 16 | 108 | 14.81 | 49 | 5.23 | 43239 |
| ACTB/G fr a | P60709 | 74 | 10 | 45 | 22.22 | 44 | 5.31 | 29799 |
| ACTB/G fr b | P60709 | 76 | 8 | 33 | 24.24 | 22 | 5.15 | 27536 |
| ACTB/G sf a | P60709 | 80 | 10 | 39 | 25.64 | 27 | 4.98 | 41242 |
| ACTB/G sf f | P60709 | 91 | 9 | 23 | 39.13 | 30 | 5.82 | 40878 |
| ACTG fr | P60709 | 60 | 4 | 9 | 44.44 | 13 | 5.34 | 33143 |
| ACTY | Q9BRB7 | 59 | 4 | 10 | 40.00 | 16 | 6.65 | 38419 |
| AGR2 | O95994 | 60 | 12 | 79 | 15.19 | 62 | 9.23 | 23125 |
| AHSA1 | O95433 | 139 | 7 | 7 | 100.00 | 27 | 5.35 | 38533 |
| AK1A1 | P14550 | 73 | 7 | 20 | 35.00 | 28 | 6.75 | 36429 |
| AK1BA a | O60218 | 76 | 8 | 49 | 16.33 | 24 | 7.04 | 33537 |
| AK1BA b | O60218 | 64 | 9 | 48 | 18.75 | 25 | 7.29 | 32465 |
| AL1A1 | P00352 | 90 | 11 | 30 | 36.67 | 25 | 6.80 | 52512 |
| ALDH2 | P05091 | 126 | 17 | 54 | 31.48 | 33 | 5.91 | 70575 |
| ALBU | P02768 | 80 | 17 | 95 | 17.89 | 27 | 5.81 | 52670 |
| ALDOA a | P04075 | 158 | 21 | 71 | 29.58 | 64 | 7.74 | 37301 |
| ALDOA b | P04075 | 65 | 13 | 51 | 25.49 | 37 | 8.00 | 37191 |
| ALDR a | P15121 | 153 | 17 | 142 | 11.97 | 38 | 6.75 | 32273 |
| ALDR b | P15121 | 153 | 17 | 142 | 11.97 | 38 | 6.78 | 32369 |
| **Abbreviated**  **Name** | **Uniprot**  **AC** | **Mowse**  **Score** | **Number of mass**  **values matched** | **Number of mass**  **values searched** | **% Masses**  **Matched** | **Seq.**  **Coverage**  **(%)** | **pI**  **exp.** | **MW (Da)**  **exp.** |
| AMPL | P28838 | 305 | 25 | 79 | 31.65 | 56 | 6.75 | 53950 |
| AN32A | P39687 | 97 | 10 | 32 | 31.25 | 30 | 4.05 | 26344 |
| ANX11 | P50995 | 133 | 26 | 84 | 30.95 | 41 | 6.87 | 53306 |
| ANXA1 | P04083 | 216 | 19 | 41 | 46.34 | 52 | 6.84 | 33836 |
| ANXA1 a | P04083 | 89 | 11 | 37 | 29.73 | 44 | 6.68 | 32465 |
| ANXA1 sf1 | P04083 | 108 | 9 | 27 | 33.33 | 27 | 7.28 | 27036 |
| ANXA1 sf2 | P04083 | 165 | 19 | 55 | 34.55 | 58 | 7.72 | 26510 |
| ANXA2 | P07355 | 78 | 17 | 93 | 18.28 | 41 | 7.30 | 32178 |
| ANXA2 a | P07355 | 66 | 5 | 8 | 62.5 | 15 | 6.89 | 32369 |
| ANXA2 sf | P07355 | 57 | 9 | 42 | 21.43 | 20 | 6.87 | 27337 |
| ANXA4 a | P09525 | 77 | 21 | 137 | 15.33 | 50 | 5.30 | 33736 |
| ANXA5 | P08758 | 162 | 18 | 45 | 40.00 | 51 | 4.84 | 29799 |
| ANXA5 b | P08758 | 213 | 20 | 38 | 52.63 | 64 | 4.91 | 29623 |
| ARP3 | P61158 | 63 | 16 | 121 | 13.22 | 30 | 5.03 | 24417 |
| APOA1 a | P02647 | 128 | 11 | 27 | 40.74 | 46 | 5.12 | 24198 |
| APOA1 b | P02647 | 148 | 19 | 70 | 27.14 | 53 | 5.58 | 48279 |
| ARPC5 | O15511 | 129 | 9 | 13 | 69.23 | 48 | 5.48 | 24596 |
| ATP5H | O75947 | 60 | 5 | 16 | 31.25 | 40 | 5.14 | 21692 |
| ATPA | Q53XX6 | 104 | 18 | 70 | 25.71 | 38 | 7.89 | 54438 |
| ATPB | P06576 | 175 | 25 | 48 | 52.08 | 46 | 4.90 | 50351 |
| ATPB a | P06576 | 239 | 28 | 63 | 44.44 | 53 | 4.97 | 47703 |
| BLVRB | P30043 | 81 | 6 | 15 | 40.00 | 44 | 6.82 | 11170 |
| B2MG | P61769 | 103 | 6 | 10 | 60.00 | 45 | 7.31 | 21974 |
| C1QBP | Q07021 | 56 | 9 | 57 | 15.79 | 37 | 4.33 | 29799 |
| C1QBP a | Q07021 | 64 | 7 | 32 | 21.88 | 40 | 4.41 | 30966 |
| CAH1 | P00915 | 108 | 7 | 10 | 70.00 | 40 | 6.87 | 26344 |
| CALM | P62158 | 73 | 5 | 6 | 83.33 | 26 | 4.09 | 16826 |
| CALR | P27797 | 98 | 21 | 168 | 12.50 | 58 | 4.31 | 61039 |
| CALR a | P27797 | 119 | 14 | 45 | 31.11 | 31 | 4.31 | 53466 |
| CALU a | O43852 | 60 | 7 | 26 | 26.92 | 22 | 4.42 | 48279 |
| CALU b | O43852 | 69 | 8 | 29 | 27.59 | 27 | 4.42 | 46431 |
| CAP1 a | Q01518 | 60 | 8 | 18 | 44.44 | 19 | 6.87 | 55761 |
| CAP1 b | Q01518 | 66 | 5 | 23 | 21.74 | 15 | 7.28 | 55261 |
| CAPG a | P40121 | 63 | 8 | 19 | 42.11 | 20 | 5.80 | 38306 |
| CAPG b | P40121 | 66 | 6 | 15 | 40.00 | 13 | 5.91 | 38306 |
| CAPG c | P40121 | 101 | 7 | 7 | 100.00 | 16 | 6.09 | 36754 |
| CATA | P04040 | 72 | 11 | 43 | 25.58 | 15 | 6.97 | 59976 |
| CATA a | P04040 | 60 | 6 | 13 | 46.15 | 11 | 7.00 | 61507 |
| CATD a | P07339 | 124 | 17 | 44 | 38.64 | 41 | 5.22 | 26344 |
| CATD b | P07339 | 100 | 13 | 22 | 59.09 | 26 | 5.59 | 26262 |
| CATD c | P07339 | 84 | 6 | 8 | 75.00 | 19 | 5.41 | 27096 |
| **Abbreviated**  **Name** | **Uniprot**  **AC** | **Mowse**  **Score** | **Number of mass**  **values matched** | **Number of mass**  **values searched** | **% Masses**  **Matched** | **Seq.**  **Coverage**  **(%)** | **pI**  **exp.** | **MW (Da)**  **exp.** |
| CATD d | P07339 | 95 | 13 | 37 | 35.14 | 31 | 6.14 | 26099 |
| CAYP1 a | Q13938 | 153 | 13 | 30 | 43.33 | 58 | 4.74 | 20868 |
| CAYP1 b | Q13938 | 189 | 11 | 13 | 84.62 | 49 | 4.75 | 20958 |
| CAZA1 | P52907 | 159 | 11 | 13 | 84.62 | 39 | 5.32 | 31057 |
| CCNH | P51946 | 87 | 6 | 9 | 66.67 | 18 | 6.75 | 33736 |
| CH10 | P61604 | 101 | 6 | 10 | 60.00 | 40 | 9.18 | 11244 |
| CH10 a | P61604 | 115 | 8 | 20 | 40.00 | 65 | 8.30 | 11227 |
| CH60 a | P10809 | 71 | 7 | 18 | 38.89 | 16 | 5.04 | 61272 |
| CH60 b | P10809 | 206 | 27 | 47 | 57.45 | 46 | 5.09 | 61742 |
| CH60 c | P10809 | 74 | 12 | 37 | 32.43 | 21 | 5.14 | 61039 |
| CH60d | P10809 | 75 | 18 | 92 | 19.57 | 34 | 5.17 | 61507 |
| CLIC1 | O00299 | 97 | 13 | 55 | 23.64 | 55 | 4.94 | 25138 |
| CLIC1 b | O00299 | 127 | 11 | 23 | 47.83 | 43 | 5.02 | 25059 |
| COF1 | P23528 | 105 | 9 | 9 | 100.00 | 52 | 6.62 | 28142 |
| COF1 a | P23528 | 102 | 6 | 8 | 75.00 | 57 | 7.91 | 29075 |
| COF1 b | P23528 | 70 | 4 | 7 | 57.14 | 40 | 8.05 | 23536 |
| COF1 c | P23528 | 69 | 8 | 36 | 22.22 | 48 | 7.28 | 29113 |
| COMT | P21964 | 72 | 10 | 50 | 20.00 | 41 | 4.90 | 24344 |
| COR1A | P31146 | 103 | 8 | 10 | 80.00 | 15 | 6.09 | 48570 |
| COTL1 | Q14019 | 62 | 6 | 30 | 20.00 | 28 | 5.19 | 16549 |
| CYTA | P01040 | 102 | 5 | 6 | 83.33 | 29 | 4.92 | 10418 |
| CYTB | P04080 | 133 | 6 | 6 | 100.00 | 61 | 6.77 | 15288 |
| CYTB sf | P04080 | 87 | 3 | 3 | 100.00 | 45 | 6.85 | 12078 |
| DDAH1 | O94760 | 111 | 13 | 49 | 26.53 | 56 | 5.40 | 33736 |
| DDAH2 | O95865 | 125 | 7 | 7 | 100.00 | 31 | 5.52 | 26927 |
| DECR a | Q16698 | 135 | 13 | 33 | 39.39 | 41 | 8.68 | 25295 |
| DECR b | Q16698 | 206 | 26 | 78 | 33.33 | 70 | 9.08 | 24903 |
| DHRS2 a | Q13268 | 169 | 18 | 51 | 35.29 | 53 | 8.91 | 26008 |
| DHRS2 b | Q13268 | 140 | 16 | 53 | 30.19 | 48 | 9.08 | 26008 |
| DLDH | P09622 | 173 | 12 | 17 | 70.59 | 28 | 6.81 | 54112 |
| DOPD | P30046 | 89 | 14 | 18 | 77.78 | 37 | 7.30 | 11262 |
| DOPD a | P30046 | 60 | 4 | 11 | 36.36 | 36 | 7.28 | 11253 |
| ECHM | P30084 | 60 | 9 | 54 | 16.67 | 32 | 6.01 | 27207 |
| ECI1 | P42126 | 61 | 6 | 19 | 31.58 | 21 | 6.11 | 27609 |
| EF1B | P24534 | 171 | 10 | 10 | 100.00 | 46 | 4.50 | 24748 |
| EF2 | P13639 | 67 | 13 | 41 | 31.71 | 17 | 6.86 | 112134 |
| EF2 fr | P13639 | 68 | 20 | 88 | 22.73 | 23 | 5.64 | 38080 |
| EFTU | P49411 | 63 | 12 | 56 | 21.43 | 24 | 6.76 | 45877 |
| EIF3B | P55884 | 82 | 10 | 18 | 55.56 | 14 | 5.35 | 34543 |
| EIF3I | Q13347 | 105 | 10 | 26 | 38.46 | 34 | 5.39 | 35369 |
| ENOA a | P06733 | 103 | 14 | 37 | 37.84 | 36 | 6.29 | 47560 |
| **Abbreviated**  **Name** | **Uniprot**  **AC** | **Mowse**  **Score** | **Number of mass**  **values matched** | **Number of mass**  **values searched** | **% Masses**  **Matched** | **Seq.**  **Coverage**  **(%)** | **pI**  **exp.** | **MW (Da)**  **exp.** |
| ENOA b | P06733 | 60 | 18 | 133 | 13.53 | 36 | 6.60 | 47703 |
| ENOA c | P06733 | 96 | 15 | 53 | 28.30 | 43 | 6.68 | 47703 |
| ENOA d | P06733 | 75 | 13 | 43 | 30.23 | 35 | 6.78 | 47703 |
| ENOA e | P06733 | 95 | 11 | 21 | 52.38 | 29 | 6.88 | 47703 |
| ENOA sf a | P06733 | 81 | 7 | 23 | 30.43 | 18 | 6.00 | 36754 |
| ENOA sf b | P06733 | 84 | 8 | 20 | 40.00 | 24 | 6.85 | 41364 |
| ENOA sf c | P06733 | 140 | 14 | 21 | 66.67 | 35 | 5.76 | 35369 |
| ENOG | P09104 | 151 | 22 | 67 | 32.84 | 55 | 4.79 | 47275 |
| ENPL | P14625 | 74 | 12 | 15 | 80.00 | 17 | 4.73 | 90122 |
| ERP29 | P30040 | 158 | 12 | 12 | 100.00 | 39 | 5.73 | 27523 |
| ERP29 a | P30040 | 68 | 8 | 32 | 25.00 | 18 | 6.29 | 27869 |
| ESTD a | P10768 | 66 | 6 | 18 | 33.33 | 35 | 6.70 | 27948 |
| ESTD b | P10768 | 108 | 16 | 90 | 17.78 | 72 | 6.73 | 27036 |
| ESTD c | P10768 | 59 | 8 | 48 | 16.67 | 25 | 6.80 | 27036 |
| EZRI | P15311 | 173 | 20 | 32 | 62.50 | 30 | 6.59 | 82541 |
| FAAA | P16930 | 65 | 12 | 67 | 17.91 | 37 | 6.76 | 42104 |
| FABP4 | P15090 | 56 | 9 | 59 | 15.25 | 59 | 6.03 | 13206 |
| FABP5 | Q01469 | 127 | 9 | 9 | 100 | 57 | 6.59 | 15154 |
| FABP7 a | O15540 | 80 | 5 | 5 | 100 | 25 | 5.14 | 15787 |
| FABP7 b | O15540 | 101 | 18 | 65 | 27.69 | 93 | 5.49 | 13547 |
| FABPH | P05413 | 162 | 10 | 16 | 62.50 | 60 | 6.08 | 14075 |
| FIBB | P02675 | 97 | 17 | 45 | 37.78 | 31 | 5.76 | 37968 |
| FIBB a | P02675 | 132 | 10 | 11 | 90.91 | 21 | 5.49 | 38080 |
| FIBG | P02679 | 94 | 15 | 60 | 25.00 | 32 | 5.42 | 49009 |
| FRIL | P02792 | 105 | 10 | 32 | 31.25 | 54 | 7.97 | 14256 |
| FKB1A | P62942 | 78 | 5 | 5 | 100.00 | 41 | 5.29 | 55928 |
| FKBP4 | Q02790 | 90 | 14 | 55 | 25.45 | 25 | 5.38 | 24023 |
| FSCN1 | Q16658 | 169 | 18 | 30 | 60.00 | 29 | 6.78 | 50806 |
| FUMH | P07954 | 116 | 10 | 16 | 62.50 | 21 | 7.29 | 45466 |
| G3P a | P04406 | 99 | 16 | 76 | 21.05 | 44 | 7.33 | 32273 |
| G3P b | P04406 | 72 | 5 | 16 | 31.25 | 23 | 7.72 | 32465 |
| G3P c | P04406 | 85 | 8 | 11 | 72.73 | 26 | 8.22 | 32465 |
| G3P d | P04406 | 59 | 4 | 13 | 30.77 | 21 | 8.43 | 32369 |
| G3P e | P04406 | 65 | 9 | 38 | 23.68 | 38 | 9.06 | 32273 |
| G6PD | P11413 | 273 | 25 | 38 | 65.79 | 46 | 6.75 | 52670 |
| G6PD a | P11413 | 216 | 24 | 51 | 47.06 | 49 | 6.76 | 58528 |
| GANAB | Q14697 | 103 | 22 | 69 | 31.88 | 21 | 5.77 | 91851 |
| GDIR1 | P52565 | 71 | 7 | 31 | 22.58 | 38 | 4.88 | 23482 |
| GDIR2 | P52566 | 62 | 7 | 22 | 31.82 | 29 | 4.98 | 22991 |
| GELS | P06396 | 58 | 12 | 50 | 24.00 | 13 | 5.73 | 83174 |
| GELS a | P06396 | 449 | 33 | 33 | 100.00 | 40 | 5.63 | 83174 |
| **Abbreviated**  **Name** | **Uniprot**  **AC** | **Mowse**  **Score** | **Number of mass**  **values matched** | **Number of mass**  **values searched** | **% Masses**  **Matched** | **Seq.**  **Coverage**  **(%)** | **pI**  **exp.** | **MW (Da)**  **exp.** |
| GGCT | O75223 | 74 | 8 | 31 | 25.81 | 33 | 4.94 | 21576 |
| GID8 | Q9NWU2 | 76 | 7 | 21 | 33.33 | 19 | 4.19 | 22186 |
| GLNA | P15104 | 70 | 14 | 101 | 13.86 | 35 | 6.73 | 41242 |
| GLOD4 | Q9HC38 | 115 | 26 | 170 | 15.29 | 74 | 5.27 | 30512 |
| GMFB | P60983 | 72 | 5 | 8 | 62.50 | 21 | 4.98 | 25709 |
| GRP75 | P38646 | 278 | 28 | 38 | 73.68 | 43 | 5.30 | 72487 |
| GRP78 | P11021 | 75 | 8 | 24 | 33.33 | 15 | 4.90 | 75023 |
| GSHB | P48637 | 161 | 16 | 60 | 26.67 | 37 | 5.49 | 49009 |
| GSTO1 | P78417 | 99 | 21 | 116 | 18.10 | 60 | 6.08 | 25936 |
| GSTO1 a | P78417 | 60 | 7 | 27 | 25.93 | 26 | 5.77 | 26017 |
| GSTP1 | P09211 | 100 | 8 | 36 | 22.22 | 54 | 5.43 | 23341 |
| GSTP1 c | P09211 | 83 | 5 | 7 | 71.43 | 34 | 6.63 | 22991 |
| GUAA a | P49915 | 90 | 21 | 97 | 21.65 | 29 | 6.78 | 73885 |
| GUAA b | P49915 | 78 | 12 | 33 | 36.36 | 17 | 6.82 | 73323 |
| HBA a | P69905 | 189 | 9 | 9 | 100 | 89 | 5.72 | 11925 |
| HBA b | P69905 | 58 | 4 | 11 | 36.36 | 28 | 9.09 | 11774 |
| HBB a | P68871 | 86 | 8 | 61 | 13.11 | 55 | 7.23 | 25620 |
| HBB b | P68871 | 72 | 5 | 13 | 38.46 | 38 | 6.93 | 12710 |
| HBB c | P68871 | 187 | 10 | 12 | 83.33 | 82 | 6.79 | 10220 |
| HCD2 | Q99714 | 79 | 11 | 48 | 22.92 | 61 | 7.28 | 24491 |
| HCDH | Q16836 | 100 | 10 | 24 | 41.67 | 40 | 8.50 | 26445 |
| HDGF | P51858 | 57 | 9 | 51 | 17.65 | 30 | 4.73 | 35474 |
| HEBP2 | Q9Y5Z4 | 77 | 6 | 15 | 40.00 | 28 | 4.70 | 24861 |
| HIBCH | Q6NVY1 | 78 | 15 | 41 | 36.59 | 24 | 7.00 | 40160 |
| HIBCH a | Q6NVY1 | 82 | 8 | 15 | 53.33 | 19 | 6.88 | 37968 |
| HMGB1 | P09429 | 83 | 10 | 15 | 66.67 | 26 | 6.66 | 23623 |
| HPT a | P00738 | 59 | 10 | 37 | 27.03 | 21 | 4.92 | 40638 |
| HPT b | P00738 | 79 | 7 | 14 | 50.00 | 19 | 5.64 | 21699 |
| HS71A | P08107 | 160 | 15 | 21 | 71.43 | 30 | 5.35 | 69240 |
| HSP74 | P34932 | 101 | 16 | 38 | 42.11 | 24 | 5.01 | 102944 |
| HSP74 a | P34932 | 92 | 16 | 42 | 38.09 | 26 | 5.23 | 102944 |
| HSP7C a | P11142 | 63 | 20 | 135 | 14.81 | 34 | 5.12 | 70845 |
| HSP7C b | P11142 | 68 | 14 | 60 | 23.33 | 28 | 5.16 | 70575 |
| HSP7C c | P11142 | 57 | 11 | 50 | 22.00 | 21 | 5.23 | 70306 |
| HSP7C d | P11142 | 57 | 5 | 16 | 31.25 | 11 | 5.38 | 72765 |
| HS90A | P07900 | 61 | 17 | 81 | 20.99 | 19 | 4.87 | 80980 |
| HS90B | P08238 | 68 | 11 | 32 | 34.38 | 14 | 4.94 | 80057 |
| HSPB1a | P04792 | 79 | 5 | 5 | 100.00 | 35 | 5.46 | 26008 |
| HSPB1b | P04792 | 152 | 8 | 8 | 100.00 | 40 | 5.64 | 25620 |
| HSPB1c | P04792 | 71 | 6 | 12 | 50.00 | 32 | 5.88 | 25620 |
| HSPB1d | P04792 | 125 | 12 | 32 | 37.50 | 52 | 5.99 | 26165 |
| **Abbreviated**  **Name** | **Uniprot**  **AC** | **Mowse**  **Score** | **Number of mass**  **values matched** | **Number of mass**  **values searched** | **% Masses**  **Matched** | **Seq.**  **Coverage**  **(%)** | **pI**  **exp.** | **MW (Da)**  **exp.** |
| HSPB1e | P04792 | 78 | 5 | 8 | 62.50 | 23 | 5.92 | 24713 |
| HSPB1f | P04792 | 62 | 9 | 81 | 11.11 | 33 | 5.00 | 26086 |
| HSPB1g | P04792 | 60 | 4 | 8 | 50.00 | 15 | 5.23 | 25620 |
| HYOU1 | Q9Y4L1 | 84 | 12 | 19 | 63.16 | 20 | 5.03 | 170331 |
| IDHC | O75874 | 273 | 20 | 22 | 90.91 | 54 | 6.73 | 44012 |
| IDHC a | O75874 | 107 | 17 | 45 | 37.78 | 45 | 6.80 | 43882 |
| IF5A1 | P63241 | 71 | 7 | 31 | 22.58 | 35 | 5.03 | 27822 |
| IF6 | P56537 | 132 | 8 | 10 | 80.00 | 43 | 4.54 | 27289 |
| IGHG1 | P01857 | 79 | 6 | 8 | 75 | 17 | 7.62 | 89436 |
| IGHG1 a | P01857 | 138 | 9 | 11 | 81.82 | 38 | 7.61 | 50959 |
| IPYR a | Q15181 | 125 | 12 | 30 | 40.00 | 48 | 5.26 | 31427 |
| IPYR b | Q15181 | 64 | 6 | 21 | 28.57 | 23 | 5.41 | 31427 |
| IVD | P26440 | 168 | 22 | 70 | 31.43 | 47 | 7.27 | 41121 |
| K1C10 a | P13645 | 80 | 9 | 22 | 40.91 | 24 | 6.87 | 80057 |
| K1C10 e | P13645 | 103 | 23 | 109 | 21.10 | 40 | 6.67 | 63658 |
| K1C10 f | P13645 | 97 | 22 | 106 | 20.75 | 40 | 6.07 | 63174 |
| K1C18 | P05783 | 77 | 10 | 27 | 37.04 | 16 | 5.13 | 46292 |
| K1C19 | P08727 | 68 | 12 | 73 | 16.44 | 23 | 4.84 | 38533 |
| K1C9 | P35527 | 117 | 20 | 67 | 29.85 | 38 | 5.23 | 47846 |
| K2C7 | P08729 | 161 | 26 | 87 | 29.89 | 47 | 4.83 | 45198 |
| K2C7 a | P08729 | 57 | 10 | 50 | 20.00 | 21 | 5.35 | 54112 |
| K2C8 a | P05787 | 79 | 11 | 34 | 32.35 | 24 | 4.84 | 40160 |
| K2C8 b | P05787 | 89 | 10 | 21 | 47.62 | 20 | 4.84 | 37522 |
| K2C8 c | P05787 | 86 | 11 | 24 | 45.83 | 22 | 4.73 | 38647 |
| K2C8 f | P05787 | 90 | 17 | 69 | 24.64 | 26 | 5.20 | 48424 |
| K2C8 g | P05787 | 75 | 15 | 67 | 22.39 | 24 | 5.16 | 47846 |
| K2C8 h | P05787 | 80 | 15 | 62 | 24.19 | 24 | 5.45 | 51112 |
| KAD2 | P54819 | 91 | 13 | 64 | 20.31 | 58 | 7.46 | 27523 |
| KCTD9 | Q7L273 | 91 | 7 | 10 | 70.00 | 14 | 5.85 | 47275 |
| KCY | P30085 | 97 | 7 | 8 | 87.50 | 20 | 5.44 | 21230 |
| KPYM a | P14618 | 62 | 10 | 35 | 28.57 | 20 | 7.30 | 60574 |
| KPYM b | P14618 | 97 | 19 | 78 | 24.36 | 36 | 7.39 | 59976 |
| KPYM c | P14618 | 60 | 5 | 15 | 33.33 | 13 | 7.30 | 68976 |
| KPYM d | P14618 | 64 | 11 | 38 | 28.95 | 16 | 6.49 | 52512 |
| LAC1 | P0CG04 | 69 | 6 | 28 | 21.43 | 21 | 7.72 | 25852 |
| LDHA | P00338 | 66 | 4 | 8 | 50.00 | 17 | 8.07 | 30692 |
| LDHB | P07195 | 67 | 5 | 8 | 62.50 | 14 | 5.65 | 32369 |
| LEG1 a | P09382 | 82 | 5 | 9 | 55.56 | 31 | 4.77 | 14075 |
| LEG1 b | P09382 | 100 | 10 | 31 | 32.26 | 61 | 4.94 | 13206 |
| LEG3 a | P17931 | 68 | 5 | 26 | 19.23 | 19 | 8.12 | 27266 |
| LEG3 b | P17931 | 98 | 8 | 37 | 21.62 | 34 | 9.12 | 27619 |
| **Abbreviated**  **Name** | **Uniprot**  **AC** | **Mowse**  **Score** | **Number of mass**  **values matched** | **Number of mass**  **values searched** | **% Masses**  **Matched** | **Seq.**  **Coverage**  **(%)** | **pI**  **exp.** | **MW (Da)**  **exp.** |
| LGUL | Q04760 | 115 | 10 | 15 | 66.67 | 36 | 4.88 | 22554 |
| LMNA | P02545 | 179 | 29 | 60 | 48.33 | 34 | 6.80 | 60574 |
| LMNA fr a | P02545 | 155 | 30 | 75 | 40.00 | 41 | 5.82 | 42731 |
| LMNA fr b | P02545 | 92 | 14 | 65 | 21.54 | 19 | 6.02 | 41121 |
| LMNA fr c | P02545 | 224 | 40 | 81 | 49.38 | 46 | 5.72 | 42731 |
| LMNA fr d | P02545 | 79 | 30 | 125 | 24.00 | 32 | 5.63 | 42354 |
| LMNA fr e | P02545 | 135 | 34 | 90 | 37.78 | 36 | 5.58 | 43239 |
| LMNA fr f | P02545 | 137 | 31 | 78 | 39.74 | 36 | 5.48 | 42354 |
| LMNA fr g | P02545 | 211 | 35 | 68 | 51.47 | 38 | 5.43 | 43111 |
| LMNA fr h | P02545 | 67 | 8 | 15 | 53.33 | 12 | 5.94 | 42731 |
| MDHC | P40925 | 72 | 12 | 54 | 22.22 | 37 | 6.82 | 32465 |
| MDHM b | P40926 | 168 | 12 | 17 | 70.59 | 38 | 8.83 | 30512 |
| MIF a | P14174 | 72 | 4 | 4 | 100.00 | 21 | 7.71 | 11275 |
| MIF b | P14174 | 64 | 4 | 4 | 100.00 | 23 | 8.07 | 11310 |
| MYL6 | P60660 | 75 | 8 | 31 | 25.81 | 47 | 4.48 | 17447 |
| MYL6 a | P60660 | 65 | 7 | 16 | 43.75 | 41 | 4.36 | 19053 |
| NEDD8 | Q15843 | 89 | 5 | 7 | 71.43 | 39 | 5.82 | 20801 |
| NDKA | P15531 | 66 | 4 | 6 | 66.67 | 25 | 9.06 | 30117 |
| NDKB | P22392 | 90 | 9 | 28 | 32.14 | 59 | 6.78 | 38193 |
| NDUAA | O95299 | 127 | 9 | 11 | 81.82 | 22 | 6.07 | 7805 |
| NPM a | P06748 | 62 | 6 | 12 | 50.00 | 23 | 4.79 | 34137 |
| NPM fr | P06748 | 76 | 8 | 32 | 25.00 | 33 | 4.45 | 21699 |
| NPM fr a | P06748 | 95 | 8 | 9 | 88.89 | 26 | 4.74 | 22447 |
| NPM fr b | P06748 | 58 | 7 | 25 | 28.00 | 25 | 4.70 | 25709 |
| NTF2 | P61970 | 74 | 7 | 19 | 36.84 | 53 | 4.80 | 11240 |
| PA2G4 | Q9UQ80 | 87 | 8 | 12 | 66.67 | 16 | 6.63 | 45198 |
| PARK7 a | Q99497 | 92 | 11 | 41 | 26.83 | 66 | 5.33 | 22369 |
| PARK7 b | Q99497 | 70 | 9 | 50 | 18.00 | 59 | 5.45 | 22369 |
| PARK7 c | Q99497 | 80 | 11 | 45 | 24.44 | 34 | 5.78 | 22339 |
| PARK7 d | Q99497 | 67 | 7 | 35 | 20.00 | 44 | 6.12 | 22247 |
| PDC6I | Q8WUM4 | 137 | 16 | 22 | 72.73 | 22 | 6.60 | 89436 |
| PDIA1 | P07237 | 185 | 24 | 71 | 33.80 | 54 | 4.76 | 57116 |
| PDIA3 a | P30101 | 219 | 15 | 15 | 100.00 | 31 | 5.56 | 54275 |
| PDIA3 b | P30101 | 69 | 13 | 64 | 20.31 | 26 | 5.70 | 54275 |
| PDIA3 c | P30101 | 150 | 24 | 72 | 33.33 | 49 | 5.81 | 53466 |
| PDIA3 d | P30101 | 150 | 23 | 59 | 38.98 | 36 | 5.96 | 53146 |
| PDIA3 e | P30101 | 64 | 10 | 31 | 32.26 | 18 | 5.47 | 56774 |
| PDXK | O00764 | 60 | 7 | 32 | 21.88 | 36 | 5.63 | 33636 |
| PEBP1 a | P30086 | 121 | 10 | 30 | 33.33 | 52 | 6.88 | 21253 |
| PEBP1 b | P30086 | 98 | 6 | 9 | 66.67 | 39 | 7.36 | 21003 |
| PFD2 | Q9UHV9 | 166 | 12 | 16 | 75.00 | 52 | 6.12 | 25709 |
| **Abbreviated**  **Name** | **Uniprot**  **AC** | **Mowse**  **Score** | **Number of mass**  **values matched** | **Number of mass**  **values searched** | **% Masses**  **Matched** | **Seq.**  **Coverage**  **(%)** | **pI**  **exp.** | **MW (Da)**  **exp.** |
| PGAM1 a | P18669 | 97 | 17 | 124 | 13.71 | 77 | 6.75 | 27785 |
| PGAM1 b | P18669 | 107 | 9 | 34 | 26.47 | 43 | 6.84 | 27869 |
| PGK1 a | P00558 | 122 | 9 | 9 | 100.00 | 25 | 7.31 | 42984 |
| PGK1 b | P00558 | 59 | 13 | 81 | 16.05 | 33 | 7.66 | 43239 |
| PGK1 c | P00558 | 73 | 10 | 23 | 43.48 | 28 | 8.10 | 43239 |
| PGM1 | P36871 | 84 | 13 | 41 | 31.71 | 18 | 6.66 | 57818 |
| PHB | P35232 | 192 | 18 | 51 | 35.29 | 68 | 5.39 | 26721 |
| PHS | P61457 | 101 | 7 | 20 | 35 | 61 | 6.63 | 10621 |
| PLPP | Q96GD0 | 59 | 6 | 28 | 21.43 | 22 | 5.80 | 35579 |
| PNPH a | P00491 | 100 | 21 | 89 | 23.60 | 66 | 6.70 | 26099 |
| PNPH b | P00491 | 89 | 6 | 6 | 100.00 | 24 | 6.66 | 27609 |
| POTEE a | Q6S8J3 | 151 | 15 | 21 | 71.43 | 13 | 4.90 | 157864 |
| POTEE b | Q6S8J3 | 125 | 15 | 18 | 83.33 | 13 | 4.92 | 157864 |
| PPIA a | P62937 | 64 | 6 | 29 | 20.69 | 56 | 6.68 | 25477 |
| PPIA b | P62937 | 57 | 7 | 53 | 13.21 | 40 | 6.81 | 24166 |
| PPIA c | P62937 | 96 | 9 | 36 | 25.00 | 41 | 7.28 | 22522 |
| PPIA d | P62937 | 145 | 9 | 14 | 64.29 | 50 | 7.29 | 24166 |
| PPIA e | P62937 | 58 | 3 | 3 | 100 | 26 | 7.53 | 24380 |
| PPIB | P23284 | 126 | 23 | 108 | 21.30 | 63 | 9.25 | 20276 |
| PPIF | P30405 | 56 | 4 | 12 | 33.33 | 14 | 9.08 | 23486 |
| PRDX1 a | Q06830 | 72 | 14 | 133 | 10.53 | 73 | 6.65 | 21576 |
| PRDX1 b | Q06830 | 86 | 8 | 31 | 25.81 | 38 | 6.84 | 22462 |
| PRDX1 c | Q06830 | 150 | 12 | 31 | 38.71 | 58 | 7.30 | 23023 |
| PRDX1 d | Q06830 | 110 | 9 | 46 | 19.57 | 45 | 8.00 | 23213 |
| PRDX2 | P32119 | 66 | 10 | 60 | 16.67 | 36 | 5.40 | 21599 |
| PRDX2 a | P32119 | 69 | 10 | 55 | 18.18 | 36 | 4.97 | 20891 |
| PRDX2 b | P32119 | 78 | 18 | 241 | 7.47 | 78 | 5.14 | 20958 |
| PRDX2 c | P32119 | 99 | 5 | 5 | 100.00 | 25 | 5.23 | 21576 |
| PRDX3 | P30048 | 97 | 13 | 58 | 22.41 | 64 | 6.01 | 23482 |
| PRDX4 | Q13162 | 64 | 13 | 145 | 8.97 | 57 | 5.57 | 26322 |
| PRDX6 | P30041 | 147 | 19 | 19 | 100 | 77 | 5.52 | 24491 |
| PROF1 a | P07737 | 94 | 8 | 22 | 36.36 | 62 | 7.28 | 16613 |
| PROF1 b | P07737 | 138 | 6 | 13 | 46.15 | 60 | 8.14 | 15692 |
| PRP19 | Q9UMS4 | 122 | 8 | 8 | 100.00 | 12 | 6.52 | 35369 |
| PRS10 | P62333 | 116 | 16 | 144 | 11.11 | 40 | 7.28 | 43111 |
| PRS6A | P17980 | 107 | 12 | 29 | 41.38 | 31 | 5.00 | 49601 |
| PRS6B | P43686 | 63 | 7 | 28 | 25.00 | 14 | 4.93 | 47846 |
| PRS7 | P35998 | 60 | 6 | 12 | 50.00 | 15 | 5.72 | 51112 |
| PRS8 | P62195 | 105 | 5 | 8 | 62.50 | 15 | 7.27 | 45466 |
| PSA1 | P25786 | 78 | 9 | 50 | 18.00 | 30 | 6.15 | 29536 |
| PSA2 | P25787 | 90 | 10 | 173 | 5.78 | 57 | 6.87 | 24491 |
| **Abbreviated**  **Name** | **Uniprot**  **AC** | **Mowse**  **Score** | **Number of mass**  **values matched** | **Number of mass**  **values searched** | **% Masses**  **Matched** | **Seq.**  **Coverage**  **(%)** | **pI**  **exp.** | **MW (Da)**  **exp.** |
| PSA3 | P25788 | 61 | 10 | 74 | 13.51 | 38 | 5.04 | 27351 |
| PSA4 | P25789 | 62 | 10 | 50 | 20.00 | 26 | 7.30 | 26593 |
| PSA5 | P28066 | 65 | 5 | 12 | 41.67 | 31 | 4.72 | 27207 |
| PSA6 | P60900 | 86 | 10 | 36 | 27.78 | 35 | 6.67 | 27125 |
| PSA7 | O14818 | 92 | 11 | 45 | 24.44 | 44 | 8.60 | 27869 |
| PSB3 | P49720 | 68 | 7 | 22 | 31.82 | 38 | 6.15 | 24417 |
| PSB5 | P28074 | 248 | 15 | 17 | 88.24 | 36 | 8.98 | 21646 |
| PSB6 | P28072 | 63 | 6 | 44 | 13.64 | 29 | 4.74 | 22709 |
| PSME1 | Q06323 | 71 | 9 | 27 | 33.33 | 31 | 5.68 | 26676 |
| PUR9 | P31939 | 87 | 12 | 32 | 37.50 | 27 | 6.73 | 63902 |
| RAB18 | Q9NP72 | 61 | 6 | 21 | 28.57 | 20 | 6.04 | 21699 |
| RABP2 | P29373 | 162 | 11 | 12 | 91.67 | 47 | 5.40 | 16826 |
| RABP2 a | P29373 | 67 | 11 | 97 | 11.34 | 64 | 5.35 | 12233 |
| RB11B | Q15907 | 63 | 8 | 47 | 17.02 | 34 | 5.60 | 21414 |
| RACK1 | P63244 | 106 | 12 | 35 | 34.28 | 31 | 7.00 | 30332 |
| RACK1 a | P63244 | 62 | 6 | 17 | 35.29 | 18 | 7.30 | 30242 |
| RET1 | P09455 | 86 | 5 | 22 | 22.73 | 36 | 5.00 | 15288 |
| RLA0 | P05388 | 118 | 15 | 63 | 23.81 | 55 | 6.10 | 32273 |
| RL40 a | P62987 | 174 | 11 | 12 | 91.67 | 92 | 5.23 | 8008 |
| RL40 b | P62987 | 125 | 6 | 7 | 85.71 | 72 | 6.89 | 8594 |
| RM12 | P52815 | 188 | 11 | 13 | 84.62 | 63 | 5.03 | 21809 |
| ROA1 | P09651 | 61 | 11 | 55 | 20.00 | 26 | 7.29 | 22156 |
| ROA1 a | P09651 | 100 | 12 | 27 | 28.57 | 38 | 7.30 | 21322 |
| ROA1 b | P09651 | 68 | 6 | 21 | 28.57 | 38 | 7.12 | 20507 |
| ROA2 | P22626 | 67 | 7 | 24 | 29.17 | 20 | 6.79 | 20436 |
| ROA2 a | P22626 | 120 | 10 | 18 | 55.56 | 34 | 6.67 | 22369 |
| RRBP1 | Q9P2E9 | 66 | 17 | 46 | 36.96 | 13 | 4.76 | 82857 |
| RSSA | P08865 | 270 | 19 | 20 | 95.00 | 60 | 4.76 | 37301 |
| RSSA a | P08865 | 97 | 9 | 19 | 47.37 | 35 | 4.78 | 41242 |
| RT22 | P82650 | 86 | 6 | 15 | 40.00 | 20 | 6.65 | 34543 |
| RUVB1 | Q9Y265 | 73 | 7 | 15 | 46.67 | 25 | 6.70 | 50959 |
| S100P | P31948 | 66 | 4 | 5 | 80.00 | 20 | 4.74 | 8568 |
| S10A2 | P29034 | 161 | 15 | 18 | 83.33 | 38 | 4.68 | 11109 |
| S10A4 | P26447 | 129 | 10 | 16 | 62.50 | 51 | 5.46 | 10418 |
| S10A6 a | P06703 | 68 | 4 | 5 | 80.00 | 40 | 4.82 | 9223 |
| S10A6 b | P06703 | 77 | 4 | 4 | 100.00 | 28 | 4.98 | 8989 |
| S10A7 | P31151 | 80 | 5 | 8 | 62.50 | 39 | 6.26 | 11332 |
| S10A7 a | P31151 | 74 | 7 | 12 | 58.33 | 35 | 5.49 | 11292 |
| S10A8 | P05109 | 83 | 10 | 44 | 22.73 | 54 | 6.87 | 11170 |
| S10A9 | P06702 | 84 | 4 | 5 | 80.00 | 37 | 5.69 | 14075 |
| S10AB | P31949 | 82 | 5 | 5 | 100.00 | 42 | 4.87 | 11135 |
| **Abbreviated**  **Name** | **Uniprot**  **AC** | **Mowse**  **Score** | **Number of mass**  **values matched** | **Number of mass**  **values searched** | **% Masses**  **Matched** | **Seq.**  **Coverage**  **(%)** | **pI**  **exp.** | **MW (Da)**  **exp.** |
| S10AB a | P31949 | 127 | 11 | 11 | 100.00 | 61 | 5.19 | 11157 |
| S10AB b | P31949 | 100 | 6 | 6 | 100.00 | 46 | 5.95 | 11126 |
| S10AD | Q99584 | 226 | 13 | 19 | 68.42 | 89 | 5.27 | 11279 |
| S10AD b | Q99584 | 79 | 4 | 4 | 100.00 | 34 | 5.69 | 11301 |
| S10AG | Q96FQ6 | 92 | 6 | 14 | 42.86 | 59 | 6.67 | 11227 |
| SBP1 | Q13228 | 114 | 12 | 23 | 52.17 | 26 | 6.02 | 52198 |
| SCRN1 | Q12765 | 105 | 16 | 75 | 21.33 | 37 | 4.72 | 51266 |
| SH3L1 a | O75368 | 62 | 4 | 31 | 12.90 | 40 | 5.03 | 11292 |
| SH3L1 b | O75368 | 105 | 10 | 66 | 15.15 | 75 | 5.16 | 12873 |
| SH3L3 | Q9H299 | 64 | 5 | 15 | 33.33 | 36 | 4.75 | 11118 |
| SH3L3 a | Q9H299 | 114 | 6 | 9 | 66.67 | 47 | 4.83 | 11139 |
| SIAS | Q9NR45 | 65 | 6 | 13 | 46.15 | 25 | 6.76 | 43882 |
| SODC a | P00441 | 58 | 3 | 7 | 42.86 | 53 | 5.36 | 28567 |
| SODC b | P00441 | 96 | 5 | 6 | 83.33 | 42 | 5.64 | 27513 |
| SODM a | P04179 | 102 | 7 | 35 | 20.00 | 38 | 6.84 | 22803 |
| SODM b | P04179 | 62 | 6 | 24 | 25.00 | 31 | 7.23 | 22803 |
| STMN1 | P16949 | 102 | 5 | 5 | 100.00 | 41 | 6.75 | 64638 |
| STIP1 | P31948 | 145 | 15 | 22 | 68.18 | 30 | 5.63 | 27822 |
| SYDC | P14868 | 60 | 12 | 70 | 17.14 | 31 | 5.06 | 11187 |
| SYWC | P23381 | 72 | 9 | 63 | 14.29 | 63 | 6.09 | 9585 |
| SUMO1 | P63165 | 78 | 4 | 4 | 100.00 | 22 | 6.69 | 64886 |
| SUMO4 | Q6EEV6 | 60 | 4 | 11 | 36.36 | 35 | 6.00 | 51112 |
| TAGL | Q01995 | 67 | 7 | 27 | 25.93 | 39 | 6.61 | 25477 |
| TAGL sf | Q01995 | 138 | 13 | 36 | 36.11 | 56 | 6.74 | 18556 |
| TAGL2 sf | P37802 | 57 | 7 | 18 | 28.89 | 33 | 5.49 | 15287 |
| TAGL2 sf a | P37802 | 72 | 9 | 28 | 32.14 | 39 | 6.72 | 16843 |
| TALDO | P37837 | 60 | 8 | 35 | 22.86 | 25 | 6.59 | 33340 |
| TBA1A a | Q71U36 | 56 | 7 | 25 | 28.00 | 21 | 4.90 | 53788 |
| TBA1A b | Q71U36 | 80 | 8 | 19 | 42.11 | 25 | 4.94 | 53950 |
| TBA1A c | Q71U36 | 121 | 11 | 21 | 52.38 | 29 | 5.01 | 53627 |
| TBA1A fr | Q71U36 | 62 | 7 | 21 | 33.33 | 16 | 4.90 | 11248 |
| TBB5 | P07437 | 120 | 11 | 14 | 78.57 | 22 | 4.90 | 37081 |
| TBB5 a | P07437 | 75 | 12 | 28 | 42.86 | 22 | 4.79 | 51266 |
| TBB5 b | P07437 | 77 | 12 | 39 | 30.77 | 26 | 4.83 | 50959 |
| TBB5 fr | P07437 | 78 | 11 | 30 | 36.67 | 24 | 5.03 | 31427 |
| TCPA | P17987 | 59 | 6 | 11 | 54.55 | 13 | 5.80 | 60343 |
| TCPB | P78371 | 67 | 7 | 14 | 50.00 | 13 | 6.36 | 54275 |
| TCPE | P48643 | 85 | 9 | 38 | 23.68 | 23 | 5.35 | 60574 |
| TCPG | P49368 | 103 | 20 | 70 | 28.57 | 35 | 6.46 | 63902 |
| TCPZ | P40227 | 161 | 19 | 38 | 50.00 | 43 | 6.73 | 55427 |
| TCTP | P13693 | 97 | 11 | 26 | 42.31 | 40 | 4.75 | 21529 |
| **Abbreviated**  **Name** | **Uniprot**  **AC** | **Mowse**  **Score** | **Number of mass**  **values matched** | **Number of mass**  **values searched** | **% Masses**  **Matched** | **Seq.**  **Coverage**  **(%)** | **pI**  **exp.** | **MW (Da)**  **exp.** |
| TERA a | P55072 | 144 | 32 | 124 | 25.81 | 46 | 5.07 | 87744 |
| TERA b | P55072 | 57 | 10 | 25 | 40.00 | 14 | 5.25 | 85429 |
| THIO a | P10599 | 124 | 6 | 6 | 100.00 | 72 | 4.76 | 11774 |
| THIO b | P10599 | 100 | 6 | 6 | 100.00 | 66 | 4.82 | 11774 |
| THTM | P25325 | 79 | 8 | 51 | 15.69 | 35 | 6.59 | 25295 |
| TIPRL | O75663 | 111 | 7 | 9 | 77.78 | 17 | 6.05 | 31149 |
| TPD52 | P55327 | 93 | 9 | 38 | 23.68 | 55 | 4.81 | 26165 |
| TPIS a | P60174 | 67 | 7 | 29 | 24.14 | 30 | 5.74 | 22897 |
| TPIS b | P60174 | 83 | 9 | 42 | 21.43 | 52 | 4.76 | 24564 |
| TPIS c | P60174 | 58 | 5 | 33 | 15.15 | 28 | 6.12 | 26165 |
| TPIS d | P60174 | 96 | 9 | 43 | 20.93 | 41 | 6.72 | 26561 |
| TPIS e | P60174 | 99 | 9 | 41 | 21.95 | 42 | 6.80 | 26402 |
| TPIS a sf | P60174 | 56 | 4 | 7 | 57.14 | 19 | 6.86 | 26561 |
| TPM1 | P09493 | 92 | 17 | 79 | 21.52 | 36 | 4.74 | 31149 |
| TPM1 a | P09493 | 74 | 12 | 62 | 19.35 | 24 | 4.73 | 33736 |
| TPM2 a | P07951 | 85 | 11 | 18 | 61.11 | 20 | 4.72 | 44404 |
| TPM4 a | P67936 | 132 | 16 | 47 | 34.04 | 37 | 4.71 | 29976 |
| TPM4 b | P67936 | 105 | 9 | 20 | 45.00 | 24 | 4.73 | 29976 |
| TRFE | P02787 | 103 | 15 | 33 | 45.45 | 23 | 6.72 | 79448 |
| TSTD1 | Q8NFU3 | 106 | 5 | 5 | 100 | 31 | 5.45 | 11148 |
| TTHY | P02766 | 74 | 4 | 9 | 44.44 | 48 | 5.41 | 18720 |
| TTHY a | P02766 | 62 | 3 | 5 | 60.00 | 39 | 5.15 | 18886 |
| TYB4 | P62328 | 121 | 7 | 9 | 77.78 | 93 | 4.76 | 11231 |
| TYB4 fr | P62328 | 81 | 4 | 5 | 80.00 | 68 | 4.80 | 6397 |
| TYPH | P19971 | 68 | 6 | 8 | 75.00 | 30 | 5.13 | 51420 |
| UB2V2 | Q15819 | 122 | 7 | 9 | 77.78 | 24 | 7.77 | 21391 |
| UBE2N | P61088 | 151 | 8 | 9 | 88.89 | 51 | 5.80 | 17914 |
| UCHL1 | P09936 | 181 | 12 | 18 | 66.67 | 49 | 5.29 | 25390 |
| UFC1 | Q9Y3C8 | 70 | 7 | 24 | 29.16 | 20 | 6.84 | 21483 |
| VATE1 | P36543 | 58 | 6 | 15 | 40.00 | 31 | 7.28 | 24903 |
| VATF | Q16864 | 169 | 10 | 13 | 76.92 | 68 | 4.97 | 11213 |
| VDAC1 a | P21796 | 84 | 11 | 58 | 18.97 | 42 | 8.20 | 25375 |
| VDAC1 b | P21796 | 107 | 14 | 77 | 18.18 | 65 | 9.08 | 26739 |
| VDAC2 | P45880 | 141 | 10 | 15 | 66.67 | 26 | 7.30 | 26154 |
| VIME a | P45880 | 280 | 20 | 40 | 50.00 | 54 | 4.75 | 43624 |
| VIME b | P45880 | 245 | 21 | 39 | 53.84 | 38 | 4.74 | 44799 |
| VIME c | P08670 | 180 | 24 | 47 | 51.06 | 43 | 4.72 | 37411 |
| VIME d | P08670 | 232 | 35 | 85 | 41.18 | 56 | 4.74 | 36972 |
| VIME e | P08670 | 69 | 10 | 25 | 40.00 | 18 | 4.76 | 40398 |
| VIME f | P45880 | 66 | 10 | 25 | 40.00 | 15 | 4.74 | 40878 |
| VIME fr | P08670 | 103 | 14 | 31 | 45.16 | 25 | 4.70 | 22803 |
| **Abbreviated**  **Name** | **Uniprot**  **AC** | **Mowse**  **Score** | **Number of mass**  **values matched** | **Number of mass**  **values searched** | **% Masses**  **Matched** | **Seq.**  **Coverage**  **(%)** | **pI**  **exp.** | **MW (Da)**  **exp.** |
| VINC a | P18206 | 83 | 29 | 118 | 24.58 | 30 | 5.99 | 119845 |
| VINC b | P18206 | 77 | 23 | 146 | 15.75 | 26 | 6.04 | 119845 |

**Table S1.** Catalogue of the 453 proteins identified in the breast cancer tissues. The table reports the following information: Abbreviated Names; Accession Numbers (AC) of the Swiss-Prot database; MALDI-TOF parameters and experimental isoelectric point (p*I*) and molecular weight (MW).
